# Highly synchronized cortical circuit dynamics mediate spontaneous pain in mice

**DOI:** 10.1172/JCI166408

**Published:** 2023-03-01

**Authors:** Weihua Ding, Lukas Fischer, Qian Chen, Ziyi Li, Liuyue Yang, Zerong You, Kun Hu, Xinbo Wu, Xue Zhou, Wei Chao, Peter Hu, Tewodros Mulugeta Dagnew, Daniel M. Dubreuil, Shiyu Wang, Suyun Xia, Caroline Bao, Shengmei Zhu, Lucy Chen, Changning Wang, Brian Wainger, Peng Jin, Jianren Mao, Guoping Feng, Mark T. Harnett, Shiqian Shen

**Affiliations:** 1 Department of Anesthesia, Critical Care and Pain Medicine, Massachusetts General Hospital (MGH), Harvard Medical School, Boston, Massachusetts, USA.; 2 McGovern Institute for Brain Research and Department of Brain and Cognitive Sciences, Massachusetts Institute of Technology, Cambridge, Massachusetts, USA.; 3 Department of Biostatistics, The University of Texas MD Anderson Cancer Center, Houston, Texas, USA.; 4 Department of Pathology, Tufts University School of Medicine, Medford, Massachusetts, USA.; 5 Department of Anesthesiology, Center for Shock, Trauma and Anesthesiology Research, University of Maryland School of Medicine, Baltimore, Maryland, USA.; 6 MGH/HST Martinos Center for Biomedical Imaging, Department of Radiology, MGH, Harvard Medical School, Boston, Massachusetts, USA.; 7 Department of Anesthesiology, the First Affiliate Hospital of Zhejiang University, Hangzhou, China.; 8 Department of Human Genetics, Emory University, Atlanta, Georgia, USA.

**Keywords:** Neuroscience, Therapeutics, Mouse models, Neuroimaging, Pain

## Abstract

Cortical neural dynamics mediate information processing for the cerebral cortex, which is implicated in fundamental biological processes such as vision and olfaction, in addition to neurological and psychiatric diseases. Spontaneous pain is a key feature of human neuropathic pain. Whether spontaneous pain pushes the cortical network into an aberrant state and, if so, whether it can be brought back to a “normal” operating range to ameliorate pain are unknown. Using a clinically relevant mouse model of neuropathic pain with spontaneous pain–like behavior, we report that orofacial spontaneous pain activated a specific area within the primary somatosensory cortex (S1), displaying synchronized neural dynamics revealed by intravital two-photon calcium imaging. This synchronization was underpinned by local GABAergic interneuron hypoactivity. Pain-induced cortical synchronization could be attenuated by manipulating local S1 networks or clinically effective pain therapies. Specifically, both chemogenetic inhibition of pain-related c-*Fos*–expressing neurons and selective activation of GABAergic interneurons significantly attenuated S1 synchronization. Clinically effective pain therapies including carbamazepine and nerve root decompression could also dampen S1 synchronization. More important, restoring a “normal” range of neural dynamics through attenuation of pain-induced S1 synchronization alleviated pain-like behavior. These results suggest that spontaneous pain pushed the S1 regional network into a synchronized state, whereas reversal of this synchronization alleviated pain.

## Introduction

Although the functions of cortical circuits vary dramatically between brain regions and species, general principles have been found that constrain neural dynamics to stable activity regimes. For example, sleep, quiet wakefulness, and locomotion all exhibit characteristic global electrophysiological signatures that originate from widely varying, but still healthy, states of synchrony (1–6). Within these states, cortical dynamics are broadly stable, so that finely tuned synaptic connectivity adjustments can produce adaptive changes in behavioral output. Substantial experimental (7, 8) and computational (9) work has delineated a number of mechanisms that cortical circuits use to stabilize their activity. A precise coordination of excitation and inhibition is the key component of this dynamical stability, implemented by processes including local circuit wiring rules (10), homeostatic regulation of intrinsic excitability (11), and synaptic scaling (12). It is well known that disease states exhibit abnormal cortical dynamics (4), but it is not clear how, or even if, this dysregulated activity actually drives maladaptive behavior. It is additionally unknown what specific cellular or circuit mechanisms produce altered cortical dynamics in disease states. While ectopic positive feedback loops have been implicated in epileptic seizures (13) and depression (14), it is unknown if this is a general mechanism. Although evidence points to alterations in the dynamical stability mechanisms mentioned above, the connection or connections between disease states and aberrant cortical mechanisms have been challenging to study, given the lack of effective translational models for many human disorders.

Chronic pain affects 11%–40% of all adults (15). As a subgroup of chronic pain, chronic neuropathic pain can be a devastating and life-altering disease. In a large human study of chronic neuropathic pain, less than one-third of patients reported evoked pain, including mechanical hyperalgesia and allodynia (16). Spontaneous pain, which occurs in the absence of external stimuli, represents a key aspect of human neuropathic pain with poorly understood etiology. Interestingly, antiseizure medicine such as carbamazepine is a first-line treatment for some chronic neuropathic pain conditions, such as trigeminal neuralgia (TN), suggesting that abnormal, hypersynchronous cortical activity may contribute to this disease state. Indeed, recent studies have reported an increase in the correlation between cortical neurons in the sensory cortex and evoked pain (17). This suggests that the pain state may entrain a fraction of cortical neurons, potentially leading to abnormally hypersynchronous patterns of activity that push cortical dynamics outside of their normal operating range.

Like many other human brain disorders, investigating the neural dynamics underlying chronic neuropathic pain has been difficult because of the technical challenges of reliably inducing and parametrizing animal models that reflect the human pain experience, particularly spontaneous pain. Existing studies have investigated synaptic plasticity and cortical activity patterns in evoked pain, with a particular focus on the anterior cingulate cortex (18). Additionally, recent evidence suggests that pain may also alter neural dynamics in the primary sensory cortex (S1) (17, 19). How spontaneous pain alters neural activity patterns in S1, and how these patterns may be brought back into normal operating range to ameliorate pain, are unknown.

We developed a pain model for inducing neuropathic pain via a simple impingement of the trigeminal nerve root. Mice that have undergone this procedure show hallmarks of TN, including spontaneous bouts of pain attacks, allodynia, avoidance of chewing solid food, and a range of other phenotypes that are also observed in humans. Using two-photon calcium imaging, we directly observed how cortical neural dynamics in S1 are altered by pain. Additionally, we found that this cortical synchronization was underpinned by local GABAergic interneuron hypoactivity. Pain-induced cortical synchronization could be attenuated by manipulating S1 networks or clinically effective pain therapies. Attenuation of S1 synchronization reliably alleviated pain-like behavior. Together, our results provide mechanistic insights into how neuropathic pain induces abnormal activity states in the cortex that can lead to debilitating pain behaviors including spontaneous pain.

## Results

### Robust neuronal activation is observed in the primary somatosensory cortex (S1).

**Figure 1:**
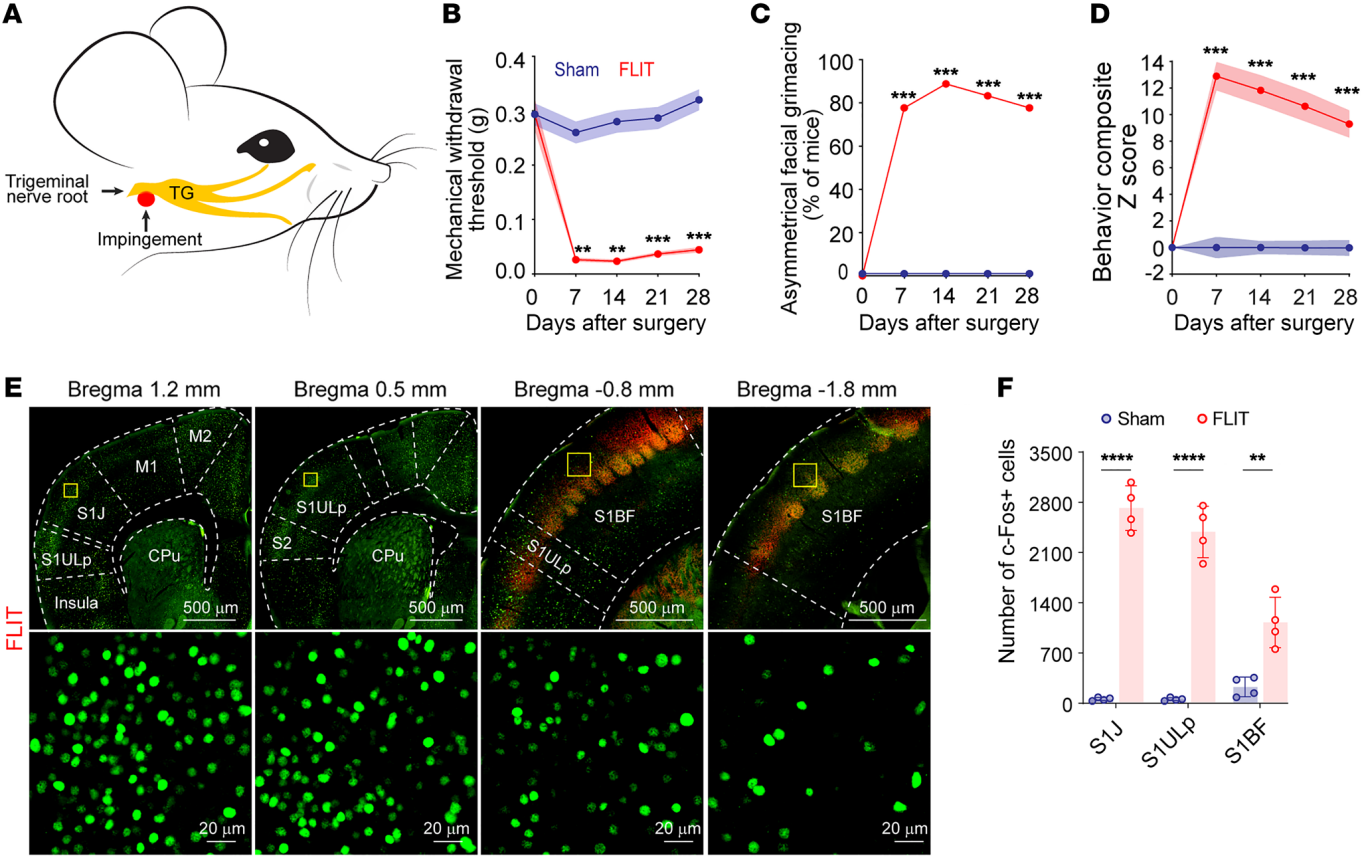
FLIT model of TN leads to robust c-*Fos* activation in the S1ULp–S1J region. (**A**) Diagram of trigeminal nerve root impingement to recapitulate human TN. Yellow structure depicts the trigeminal anatomy including the trigeminal nerve root, the trigeminal ganglion (TG), and peripheral branches; red represents the Surgifoam impingement site at the trigeminal nerve root. (**B**–**D**) Behavioral testing for the FLIT model. Mice underwent sham (*n* = 18) or FLIT (*n* = 18) surgery, followed by behavioral testing at the indicated time points. (**B**) Mechanical withdrawal threshold for von Frey filament testing (data indicate the mean ± SEM). **P < 0.01 and ***P < 0.001, by 2-way ANOVA with Bonferroni’s post hoc test for differences between groups. (**C**) Percentage of mice with asymmetrical facial grimacing behavior. Mice in the FLIT group displayed paroxysmal asymmetrical facial grimacing. ***P < 0.001, by Fisher’s exact test. (**D**) Summary quantification of behavioral tests quantified as a composite *z* score (mechanical withdrawal; grooming; body weight; length of incisors; wood weight changes; percentage of time spent eating solid chew), computed over 28 days. ***P < 0.001, by 2-way ANOVA with Bonferroni’s post hoc test differences between groups. (**E**) c-*Fos* activation in the S1ULp–S1J region after surgery. Representative tangential slices of c-*Fos* staining of FLIT-operated mice (original magnification, ×4 and ×10). Sequential slices from left to right represent coronal sections covering S1J (bregma 1.2 mm), S1ULp (bregma 0.5 mm), anterior S1BF (bregma –0.8 mm), and posterior S1BF (bregma –1.8 mm) cortex regions. Slices between bregma –0.8 mm and –1.8 mm were costained with VGLUT2 (red) to visualize barrels. Lower panels represent boxed regions of the corresponding upper panels. Scale bars: 500 μm (top row) and 20 μm (bottom row). (**F**) Quantification of c-*Fos*^+^ cells in the S1J, S1ULp, and S1BF cortex regions (*n* = 4 per group). **P < 0.01, ****P < 0.0001, by 2-way ANOVA with Bonferroni’s post hoc test to determine significant differences between groups.

In the somatosensory homunculus, the orofacial area, among all body areas, represents the largest projection within the S1 (20, 21). We therefore reasoned that an orofacial neuropathic pain model with robust spontaneous pain–like behavior would facilitate a mechanistic query of cortical substrates of pain. Human TN is a prototypic neuropathic pain condition characterized by lancinating pain, including both evoked pain and spontaneous paroxysmal pain attacks. Compelling clinical evidence indicates that trigeminal nerve root compression is one of its major causes (22). We therefore modeled TN by mimicking the human pathology of trigeminal nerve root impingement at its entry zone (23). We discovered that a natural orifice, the foramen lacerum, lies underneath the trigeminal nerve root, across species (Supplemental Figure 1A; supplemental material available online with this article; https://doi.org/10.1172/JCI166408DS1). Taking advantage of this, we applied Surgifoam to create trigeminal nerve root compression (foramen lacerum impingement of trigeminal nerve root [FLIT]) (Figure 1A and Supplemental Figure 1B). Besides mechanical allodynia (Figure 1B), the FLIT model mice demonstrated robust spontaneous behavior that was likely related to pain, including excessive spontaneous facial grooming (Supplemental Figure 1C and Supplemental Movie 1) and spontaneous paroxysmal asymmetrical facial grimacing with intermittent eye squinting, ipsilateral to the side of the nerve compression (Figure 1C, Supplemental Figure 1D, and Supplemental Movie 2). Uncontrollable facial twitching or tic-like grimacing is commonly seen in patients with TN; facial grimacing has been increasingly accepted as an indication of pain in animals (24). Spontaneous paroxysmal asymmetrical facial grimacing, therefore, may represent paroxysmal pain attacks, a key feature of human TN (23). Moreover, we assessed the functional implications of pain, including body weight gain (Supplemental Figure 1E), wood chewing (Supplemental Figure 1F), length of incisors (Supplemental Figure 1G), and solid versus soft chew preference (Supplemental Figure 1, H–J). Compared with sham control mice, the FLIT model mice had an initial phase of weight loss followed by significantly less body weight gain, overgrowth of incisors, minimal wood chewing, and avoidance of solid chew. As the trigeminal nerve is a mixed nerve and its motor innervation controls masseter muscles, we ruled out masseter muscle atrophy as the cause of pain-like behavior (Supplemental Figure 2, A–I). For a global assessment of pain, we computed composite pain scores consisting of 6 pain-related behaviors, including mechanical withdrawal thresholds, spontaneous grooming, body weight change, increase in the length of the incisors, food preference, and wood chewing activity (see Methods). This composite pain score provides a comprehensive assessment of evoked pain, spontaneous behavior, as well as a functional implication of pain (Figure 1D). Moreover, we found that FLIT model mice exhibited anxiety-like behavior (Supplemental Figure 3, A–C) and sexual dysfunction (Supplemental Figure 3, D–F), further substantiating this model’s clinical relevance. Of note, equal numbers of male and female mice were used for the FLIT model experiments, and there was no significant difference observed in their behavioral features. Hence, the behavioral data were based on a combined analysis of both sexes.

To examine the cortical activation in response to pain, c-*Fos* was immunostained in tangential slices of the brain 2 hours after FLIT or sham surgery (Supplemental Figure 4, A and B). These mice did not undergo any mechanical or thermal testing to minimize behavioral test–evoked pain or c-*Fos* expression. VGLUT2 was used as a costain to identify the barrel cortex (BF). Brain regions including the primary motor cortex (M1), the secondary motor cortex (M2), the insula, the prefrontal cortex (PFC), S1, and the secondary somatosensory cortex (S2) were compared between the FLIT and sham control mice. Among these regions, S1 displayed the most striking increase in c-*Fos*^+^ cells (Figure 1E and Supplemental Figure 4C). More important, the S1 upper lip (S1ULp) and S1 jaw (S1J) regions exhibited the most dramatic increase among all cortical regions examined. Interestingly, S1BF, bordering the S1ULp–S1J region, exhibited only moderate increases in c-*Fos*^+^ cells (Figure 1F), and these cells were primarily located at the anterolateral portion of the BF bordering the S1ULp region. When VGLUT1 and GAD67 were used to costain for excitatory and inhibitory neurons, respectively, most c-*Fos*^+^ cells were VGLUT1^+^, indicating that a majority of these cells were excitatory neurons (Supplemental Figure 4, D and E). The FLIT model, therefore, exhibited a behavioral battery with robust features of spontaneous pain, which was accompanied by distinct c-*Fos* expression in the S1ULp–S1J region, consistent with cortical activation.

### Cortical synchronization in the S1ULp–S1J region induced by pain.

**Figure 2:**
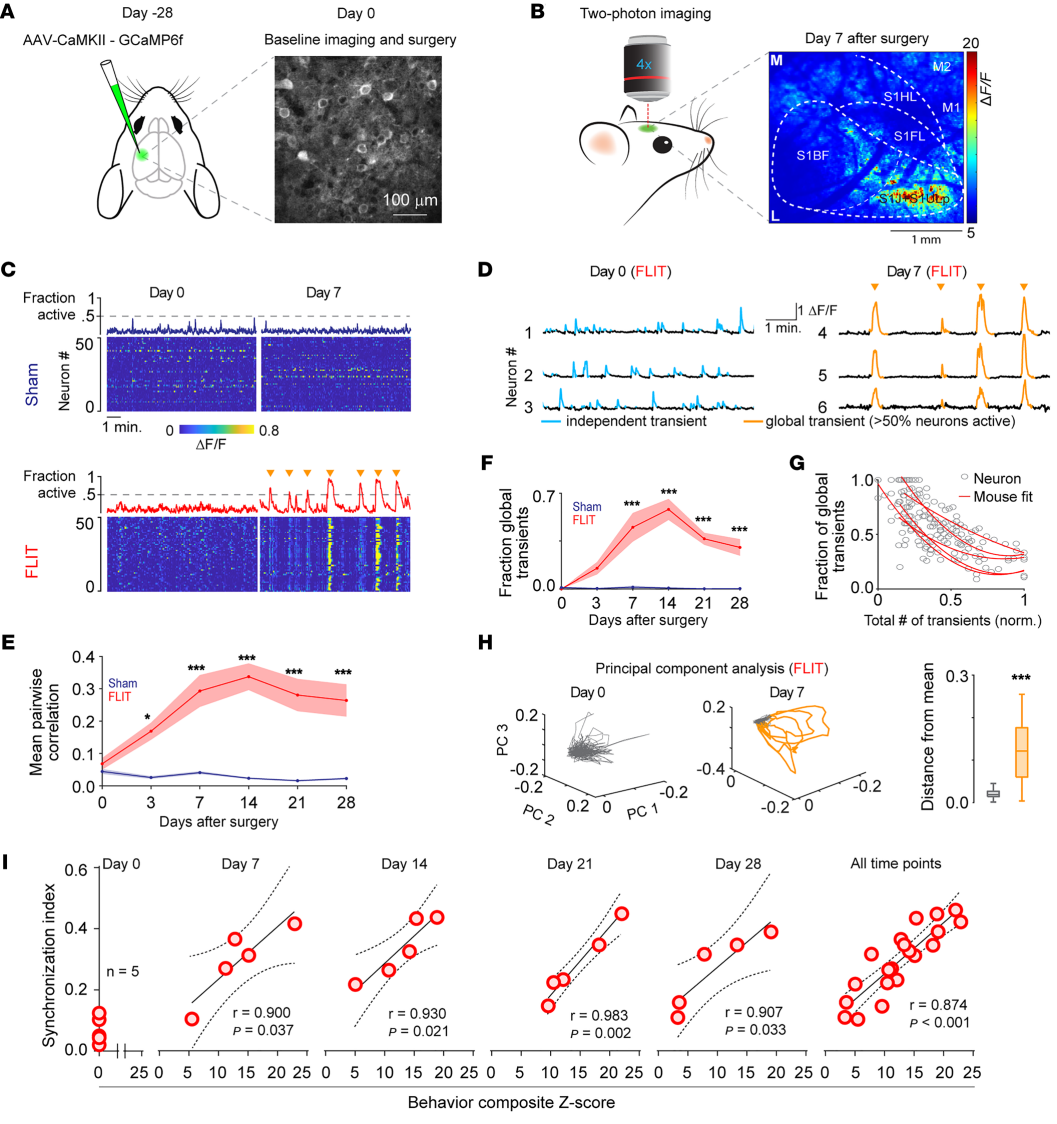
Highly synchronized S1 neural dynamics in FLIT mice. (**A**–**H**) Two-photon imaging of the anterolateral S1 cortex in awake mice (*n* = 7 sham, *n* = 5 FLIT). Images were acquired in the same field of view across all days. (**A**) Left: Schematic of AAV injection into the S1 cortex contralateral to the FLIT surgery side. Right: Representative image of GCaMP6 expression in the S1 cortex. Scale bar: 100 μm. (**B**) Robust neuronal activities in the S1ULp–S1J regions captured by wide-field two-photon calcium imaging in large cortical areas. Heatmap shows calcium activity in the imaging field 7 days after FLIT surgery. M, middle; L, lateral. Scale bar: 1 mm. (**C**) Representative heatmaps and the corresponding fraction of simultaneously active neurons for each group at days 0 and 7. Orange triangles indicate global events. (**D**) Sample neuron calcium transients from S1 of a FLIT-operated mouse at days 0 and 7. Gold traces indicate global synchronized events. (**E**) Mean pairwise correlation coefficient across days and groups. The FLIT group exhibited a significantly higher correlation. *P* < 0.001, by 2-way ANOVA versus the sham group; *P < 0.05 and ***P < 0.001, by Bonferroni’s post hoc test for pairwise comparison. (**F**) Fraction global transients across days and groups. *P* < 0.001 versus the sham group, by 2-way ANOVA; *P < 0.05 and ***P < 0.001 for comparison of global transients, by Bonferroni’s post hoc test. (**G**) Fraction of global transients per neuron (normalized to each animal). Each circle indicates 1 neuron, and each line represents the fit for 1 animal from the FLIT group. (**H**) Left and middle panels: Representative plots of neuronal trajectories using the first 3 coefficients of PCA in FLIT model mice at days 0 and 7. Activity during the global events is highlighted in gold. Right panel: Euclidean distance between the mean of the first 3 coefficients and global events (gold) versus nonglobal events (gray). ***P < 0.001, by Wilcoxon rank-sum test. (**I**) Correlation between the *z* score and the synchronization index in the FLIT model. There was a positive correlation between the *z* score and the synchronization index at the indicated time points. The solid line represents linear regression, and the dashed line represents 95% CI.

To directly assess the cortical neural dynamics associated with pain, AAV-CaMKII-GCaMP6f was microinjected into a relatively wide cortical area (2–3 mm × 3–3.4 mm) in C57BL/6 mice (Figure 2A). Animals then underwent sham or FLIT surgery, followed by intravital two-photon imaging, with both lower magnification (×4, wide-field) and higher magnification (×20). Mice were head-fixed without the use of anesthesia, as anesthetics are known to suppress cortical neural activity (25). More important, the animals were free of experimentally imposed stimuli to minimize evoked pain. On day 7 after FLIT surgery, under lower magnification, mice in the FLIT group displayed relatively higher neural activities compared with mice in the sham group, consistent with previous reports of cortical activation in response to pain (26, 27). More interestingly, neural activities among the examined cortical areas (S1, S2, M1, M2, insula, etc.) were not homogenous. Instead, they were markedly higher in a relatively small area within the S1 region (Figure 2B), which corresponded to S1ULp–S1J, the same region with robust c-*Fos* expression shown in Figure 1. We then examined this region under higher magnification at single-neuron resolution before and after induction of pain. Very surprisingly, after induction of pain, neurons in the S1ULp–S1J region started to fire synchronously in the awake and resting states, in the absence of experimentally imposed stimuli. This synchronization pattern was absent in all animals before induction of pain or in the sham group at all time points. Using 50% of the imaged neurons with simultaneous activation as a cut-off, bouts of spontaneous synchronous firing occurred once every 2–5 minutes on average (Figure 2, C and D, Supplemental Figure 5, A–C and H–J, and Supplemental Movie 3). For any imaging field, each neuron was computed against all other neurons to derive a correlation index for quantification of the synchronicity of neuronal firings. Results showed that the FLIT model had significantly higher correlation indices (Figure 2, E–H, and Supplemental Figure 5, D–G and K–M). Additionally, composite scores of pain-like behavior and S1ULp–S1J neuronal synchronization indices were plotted for correlation analysis, and we found a statistically significant correlation between these 2 parameters (Figure 2I), suggesting a plausible link between neural synchronization and pain.

### Pain-induced c-Fos–expressing neurons are responsible for synchronized neural dynamics.

**Figure 3:**
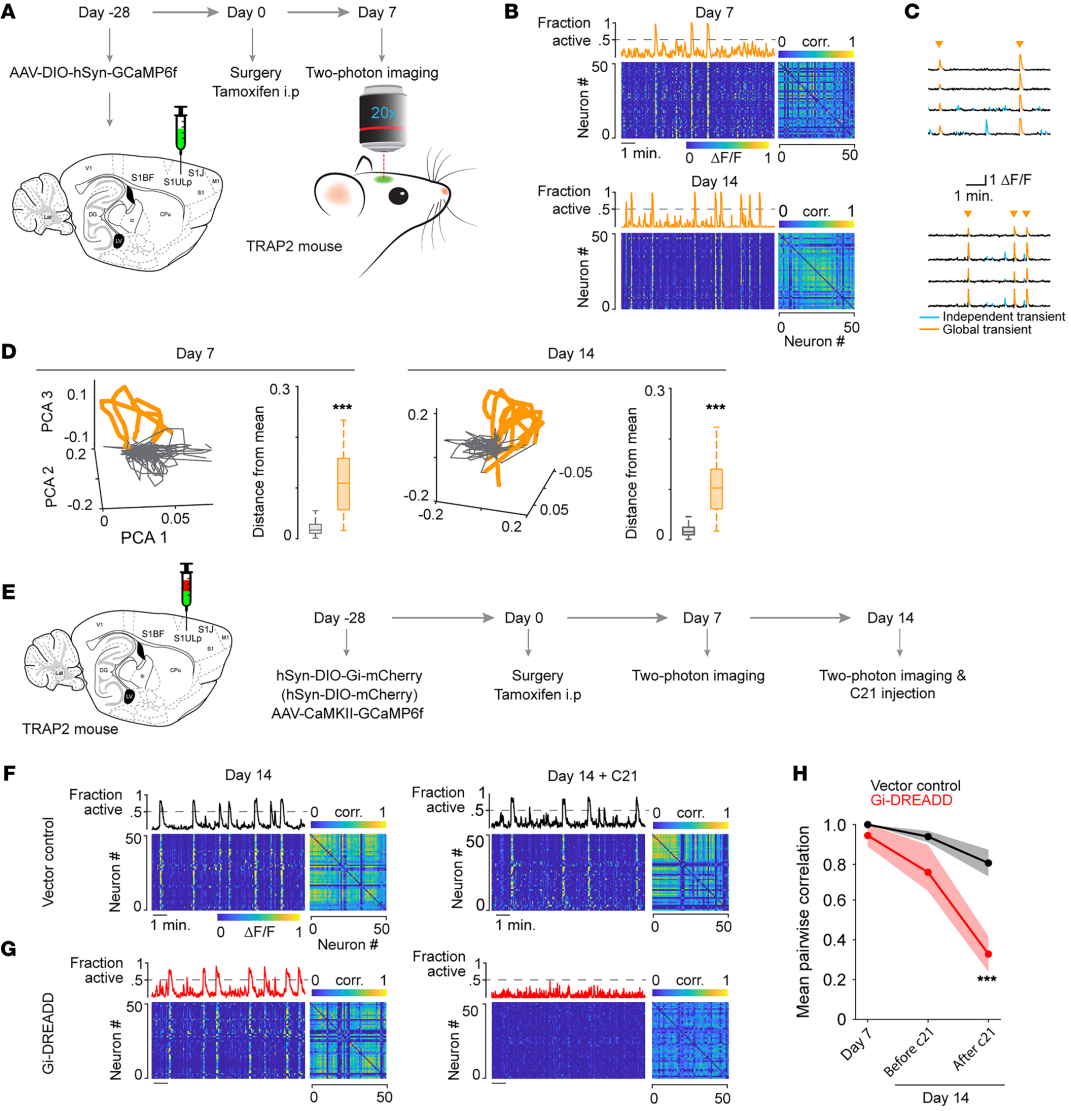
Pain-related c-*Fos*–expressing neurons drive S1 synchronization. (**A**–**D**) Neuronal synchronization captured in the S1ULp–S1J regions of a TRAP2 mouse 7 days after FLIT surgery. (**A**) Diagram and flowchart of two-photon imaging of a TRAP2 mouse (*n* = 3). (**B**) Representative heatmaps with the corresponding fraction of simultaneously active neurons and correlation matrices at days 7 and 14. Global synchronized neuron activity (>50% neurons active simultaneously) was present in TRAP2 mice that underwent FLIT surgery. (**C**) Sample neuron calcium transient traces. Gold arrowhead indicates global synchronized events. (**D**) Representative plots of neuronal trajectories using the first 3 coefficients of PCA in FLIT mice at days 7 and 14. Activity during global events is highlighted in gold. Euclidean distance between the mean of the first 3 coefficients and global events (golden) versus nonglobal events (gray). ***P < 0.001, by Wilcoxon rank-sum test. (**E**–**H**) S1ULp-S1J neuronal synchronization was subdued by inhibition of c-*Fos*–induced Gi-DREADD–expressing neurons in TRAP2 mice with TN. (**E**) Diagram and flowchart of inhibition of c-*Fos*–induced Gi-DREADD–expressing neurons in S1ULp–S1J regions of a TRAP2 mouse (*n* = 3). (**F**) Representative heatmap and correlation matrix showing neuronal synchronization present from day 7 after FLIT and tamoxifen administration. C21 administration in mice injected with a vector virus did not alter synchronization. (**G**) Representative heatmap and correlation matrix showing neuronal synchronization presented since day 7 after FLIT and tamoxifen. C21 administration to mice injected with Gi-DREADD virus suppressed neuronal synchronization. (**H**) Mean pairwise correlation coefficient across days and groups. Both groups of mice injected with vector or Gi-DREADD virus exhibited a significant correlation from day 7 after FLIT surgery, whereas C21 administration decreased the correlation in mice injected with Gi-DREADD compared with mice injected with vector virus. ***P < 0.001, by 2-way ANOVA with Bonferroni’s post hoc test for differences between vector control and Gi-DREADD. corr., correlation.

To examine the neural dynamics of pain-induced c-*Fos*–expressing neurons, Fos^2A-iCreER^ (TRAP2) mice, which express tamoxifen-inducible Cre recombinase under the control of *Fos* promoter/enhancer elements (28), were used to drive Cre-dependent GCaMP6f expression for intravital two-photon calcium imaging. Specifically, TRAP2 mice were microinjected with AAV-DIO-hSyn-GCaMP6f in the S1ULp–S1J region and then underwent FLIT or sham surgery 4 weeks later. Within 5 minutes of surgery, tamoxifen was administered intraperitoneally. A single dose of tamoxifen was able to reliably induce Cre for 4–6 hours (29). From day 7 after tamoxifen injection, intravital two-photon imaging was performed for layer 2/3 neurons in awake mice (Figure 3A) to capture the neural dynamics of c-*Fos*–expressing neurons. In mice that received FLIT surgery, we again observed strikingly synchronized neuronal activities in the S1ULp–S1J region (Figure 3, B–D), similar to the activities observed in the mice depicted in Figure 2. Synchronization of pain-related c-*Fos*–expressing neurons suggests that these neurons might be responsible for the overall S1ULp–S1J synchronization in pain. To test this, we microinjected TRAP2 mice with Cre-dependent hSyn-Gi-mCherry Gi designer receptors exclusively activated by designer drugs (Gi-DREADD) and AAV-CaMKII-GCaMP6f in the S1ULp–S1J region, followed by FLIT surgery 4 weeks later (Figure 3E). On day 7 after FLIT surgery, we observed robust S1ULp–S1J synchronization (Figure 3F), consistent with Figure 2. Tamoxifen was then administered to facilitate c-*Fos*–induced Cre expression. On day 14 after FLIT surgery, C21 was given to activate Gi-DREADD (30), S1ULp–S1J neuronal activities were imaged prior to and after C21 administration. Results showed that inhibiting these pain-induced c-*Fos*–expressing neurons largely abrogated S1ULp–S1J synchronization (Figure 3, G and H). As such, these neurons were the likely source of the synchronized S1ULp–S1J neural dynamics.

### S1ULp–S1J c-Fos–expressing neurons mediate pain-like behaviors.

**Figure 4:**
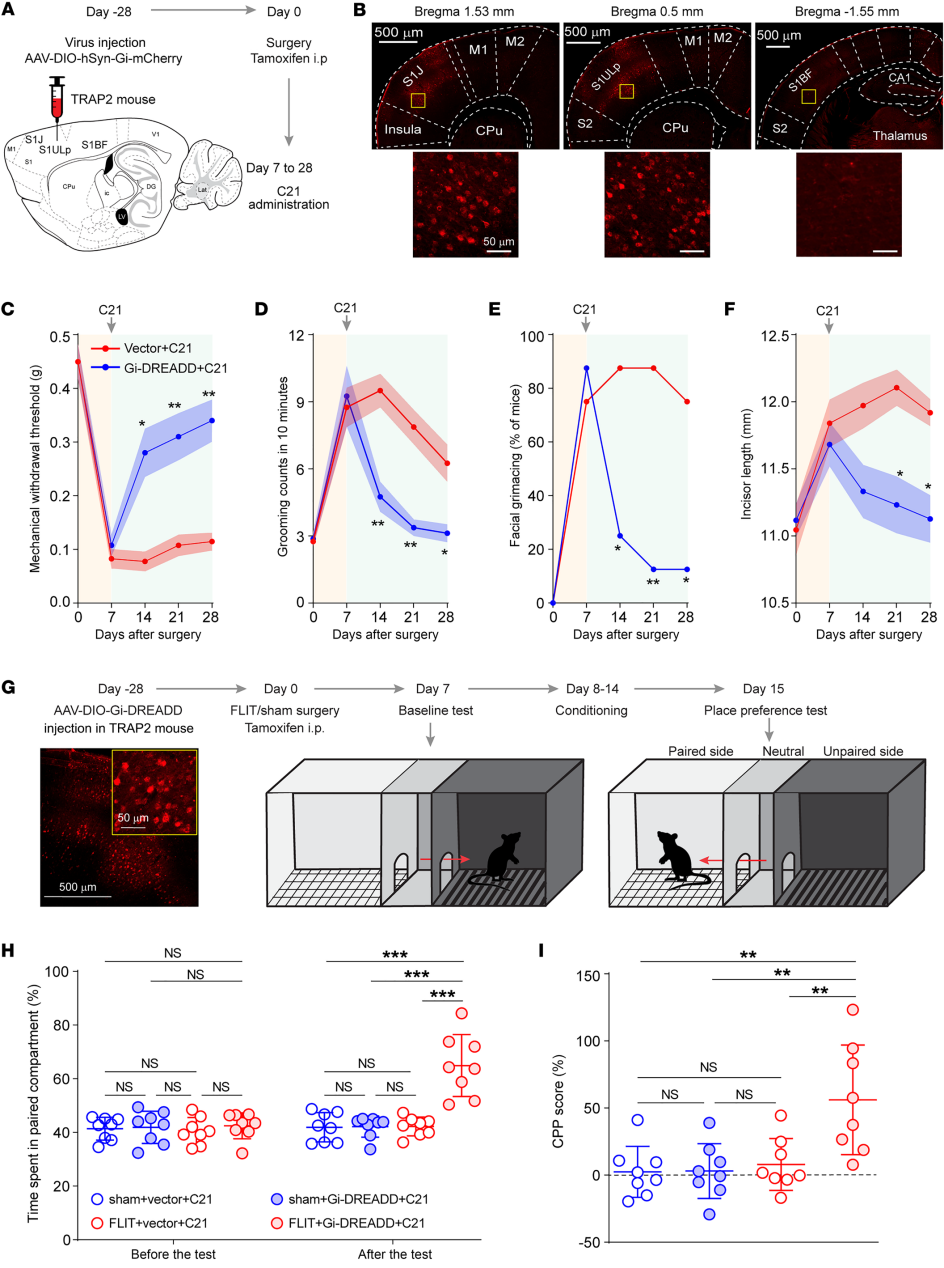
Pain-related c-*Fos*–expressing neurons are critical for pain-like behaviors. (**A**–**F**) A TRAP2 mouse was injected with AAV-DIO-hSyn-Gi-mCherry or AAV-DIO-mCherry vector in the S1ULp–S1J region at day –28 (*n* = 8 per group). FLIT surgery was performed on all mice at day 0, accompanied by tamoxifen administration. From day 7, C21 was intraperitoneally administrated twice daily. Mice were sacrificed on day 28 to obtain brain slices. (**A**) Diagram of virus injection and flowchart of the experiment timeline. (**B**) Upper panels: Representative tangential slices of S1J, S1ULp, and S1BF regions demonstrating that mCherry expression was primarily located in the S1ULp–S1J region. Lower panels represent boxed regions of the corresponding upper panels. Scale bars: 500 μm (upper panels) and 50 μm (lower panels). (**C**–**F**) Chemogenetic inhibition of c-*Fos*–induced Gi-expressing neurons led to attenuated pain-like behavior. Behavioral testing was performed at the indicated time points. At day 7, behavioral testing was performed prior to C21 administration to obtain a pretreatment baseline. *P < 0.05 and **P < 0.01, by 2-way ANOVA with Bonferroni’s post hoc test. Data indicate the mean ± SEM. (**C**) Mechanical withdrawal threshold for von Frey filament testing. (**D**) Facial grooming counts in 10 minutes. (**E**) Percentage of mice with asymmetrical facial grimaces. (**F**) Incisor length. (**G**–**I**) Chemogenetic inhibition of c-*Fos*–induced Gi-expressing neurons led to CPP behavior. TRAP2 mice were injected with either AAV-DIO-Gi-DREADD or vector virus on day –28. FLIT or sham surgery was performed on day 0, accompanied by tamoxifen administration. After a baseline test on day 7, C21 was intraperitoneally administrated twice daily, followed by conditioning and then a place preference test on day 15. **P < 0.01 and ***P < 0.001, by 2-way ANOVA with Bonferroni’s post hoc test. Data indicate the mean ± SEM. (**G**) Flowchart of CPP experiment. Representative images show c-*Fos*–induced mCherry-expressing neurons in the S1ULp–S1J region. Scale bars: 500 μm and 50 μm (enlarged inset). (**H**) Percentage of time spent in the C21-paired compartment. (**I**) CPP score.

To determine whether the c-*Fos*–expressing neurons were implicated in pain-like behaviors, TRAP2 mice underwent microinjection of Cre-dependent AAV-DIO-hSyn-Gi-mCherry (Gi-DREADD) or AAV-DIO-hSyn-mCherry (vector control) in the S1ULp–S1J region followed by FLIT surgery 4 weeks later (Figure 4, A and B). Tamoxifen was administered immediately after FLIT surgery to facilitate the induction of Cre expression. A period of 7 days was allowed for Gi-DREADD expression. On day 7, the DREADD actuator drug C21 (30) was given at a dose of 0.8 mg/kg twice daily to assess the effects of inhibiting these c-*Fos*–expressing neurons. Pain behaviors including paroxysmal asymmetrical facial grimacing were greatly reduced in mice that received Gi-DREADD but not in mice that received the vector control (Figure 4, C–F, and Supplemental Figure 6, A–C). We also assessed relief of pain by a conditional place preference test (Figure 4G), which has been increasingly used as a reliable measurement of pain relief (31). Results showed that mice that received Gi-DREADD showed a significant preference for the C21-paired chamber, but not the saline-paired chamber (Figure 4, H and I). On the other hand, mice that received the vector control did not show a preference for any chamber, supporting the idea that inhibiting c-*Fos*–expressing neurons in the S1ULp–S1J region alleviated spontaneous pain as well as other pain-related behaviors.

Although c-*Fos* expression was primarily induced in the S1ULp–S1J region, there was noticeable expression in the S1BF. To examine whether those c-*Fos*^+^ neurons in the S1BF were also implicated in spontaneous orofacial pain, the TRAP2 mice were microinjected with Gi-DREADD or the vector control in the S1BF region followed by FLIT surgery and C21 injection (Supplemental Figure 6, D and E). The results showed that twice-daily C21 injection in the Gi-DREADD group did not significantly alleviate spontaneous pain, as determined by spontaneous facial grimacing (Supplemental Figure 6, F–L).

### Local GABAergic neuron hypoactivity promotes S1 neural synchronization.

**Figure 5:**
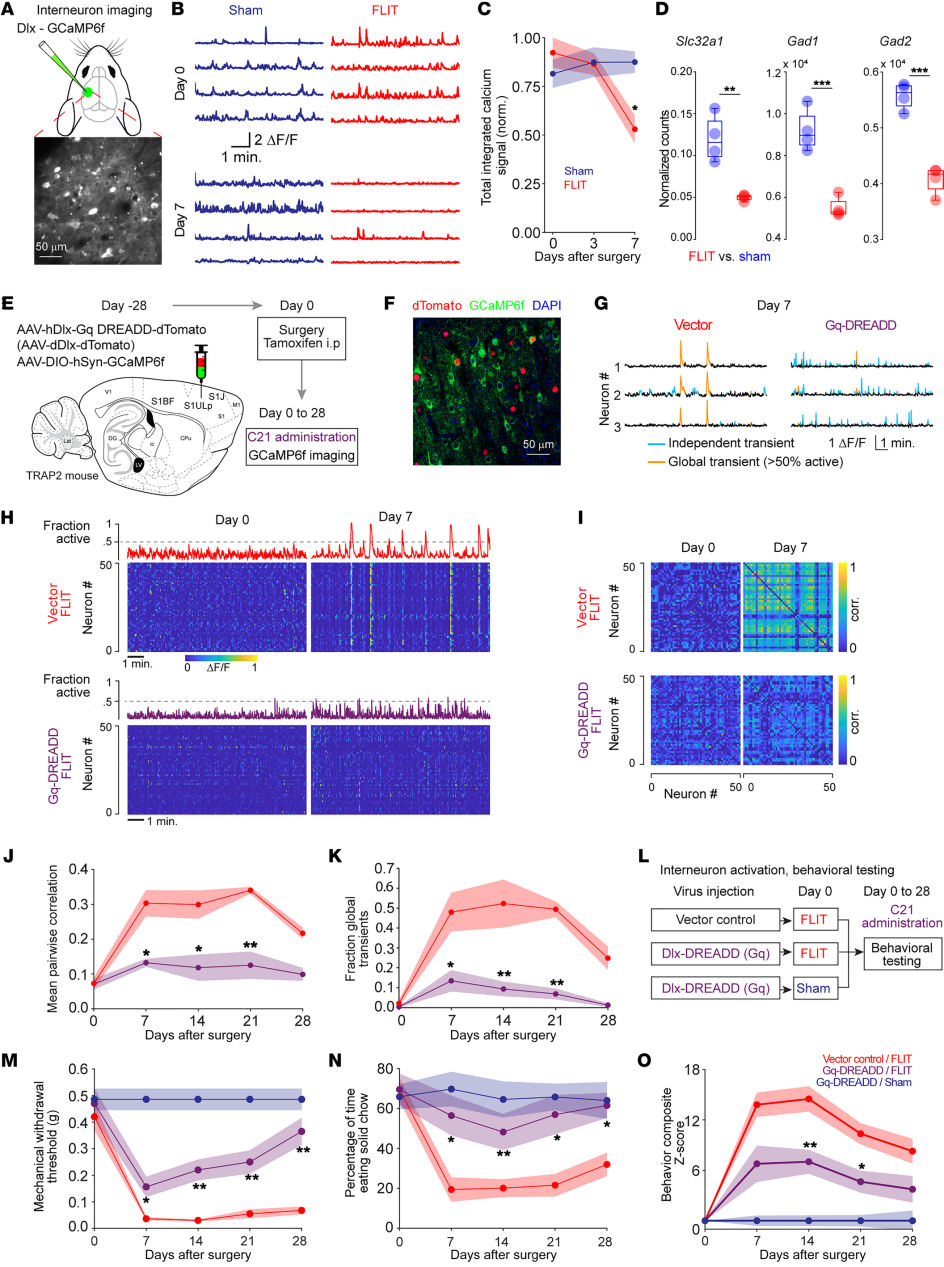
Dampening S1 synchronization through interneuron activation alleviates pain-like behavior. (**A**–**C**) Interneuron calcium imaging. The S1ULp–S1J region was injected with AAV-Dlx-GCaMP6f on day –28, followed by FLIT or sham surgery on day 0. Calcium imaging was performed at the indicated time points (*n* = 4 per group). (**A**) Diagram of S1 injection. Lower panel shows representative GCaMP6f expression. Scale bar: 50 μm. (**B**) Representative single neuron calcium dynamics tracing. (**C**) Total integrated calcium signals. *P < 0.05, by 2-way ANOVA with a post hoc Tukey-Kramer test shows significant difference between the FLIT and sham groups. (**D**) Comparison of GABAergic interneuron–related gene transcripts. **P < 0.01 and ***P < 0.001, by unpaired *t* test. (**E**–**K**) Chemogenetic activation of interneurons dampened the synchronization of c-*Fos*–induced pain-related neurons (sham *n* = 6, FLIT *n* = 5). (**E**) Diagram and flowchart of the experimental design. (**F**) Representative brain slice showing expression of dTomato and GCaMP6f within the S1ULp–S1J region. (**G**–**I**) Chemogenetic activation of interneurons decreased the synchronization of c-*Fos*–induced pain-related neurons. (**G**) Representative calcium dynamics tracing at day 7 after FLIT surgery. (**H**) Representative heatmaps and fraction of active neuron plots. (**I**) Representative correlation matrix plots. (**J**) Mean pairwise correlation at different time points after FLIT surgery. (**K**) Fraction of global transients among total transients at different time points after FLIT surgery. *P < 0.05 and **P < 0.01, by 2-way ANOVA; a post hoc Tukey-Kramer test was performed to determine the *P* value for vector/FLIT versus Gq-DREADD/FLIT. (**L**–**O**) Chemogenetic activation of interneurons alleviated pain-like behavior (*n* = 7 sham, *n* = 8 for the other groups; data indicate the mean ± SEM). (**L**) Flowchart of the experimental design. (**M**) Mechanical withdrawal threshold. (**N**) Solid food preference. (**O**) Composite *z* scores (mechanical withdrawal; grooming; body weight; incisor length; wood weight changes; and solid food preference) were computed for all groups. (**M**–**O**) *P < 0.05 and **P < 0.01, by 2-way ANOVA; a post hoc Bonferroni test was performed to determine the *P* value of vector/FLIT versus Gq-DREADD/FLIT.

Cortical GABAergic interneurons are critical components of cortical local circuits and have indispensable roles in modulating the timing, extent, and propagation of excitatory neuronal activities (32, 33). We examined GABAergic neurons in the FLIT model by taking advantage of a pan-interneuron targeting AAV-hDlx-GCaMP6f for calcium imaging (34). This tool was able to target approximately 85% of all GAD67^+^ interneurons as shown in Supplemental Figure 7, A and B). Intravital two-photon calcium imaging was performed in layer 2/3 neurons of the S1ULp–S1J region in awake mice (Figure 5A). Results showed that for the GABAergic interneurons, mice in the FLIT group displayed significantly lower total integrated calcium activities than did mice in the control group (Figure 5, B and C), suggesting hypoactivity of these local GABAergic interneurons. RNA-Seq was performed in S1ULp–S1J neurons for the FLIT and sham groups, and the results showed a dramatic decrease in the expression of GABA-related genes (Figure 5D and Supplemental Figure 7, C–E), suggesting that GABAergic interneuron hypoactivity was implicated in excitatory neuron synchronization and pain.

To directly examine the functional significance of GABAergic interneurons in synchronized neural dynamics and pain, AAV-hDlx-Gq DREADD-dTomato (Gq DREADD) was used to activate GABAergic interneurons, with AAV-dDlx-dTomato as the vector control. For imaging studies, the S1ULp–S1J region of TRAP2 mice was microinjected with a mixture of AAV-hDlx-Gq DREADD-dTomato and AAV-DIO-hSyn-GCaMP6f or a mixture of AAV-dDlx-dTomato (vector) and AAV-DIO-hSyn-GCaMP6f, followed by FLIT surgery 4 weeks later (Figure 5, E and F). Tamoxifen was administered to induce Cre expression, and C21 was injected twice daily to activate Gq DREADD. When two-photon calcium imaging was performed in awake mice, mice that received the vector control displayed robust synchronization, as shown in Figure 3. However, in mice that received Gq DREADD, the synchronization was nearly abrogated (Figure 5, D–K). Thus, activating S1ULp–S1J GABAergic neurons abrogated neuronal synchronization in S1.

To assess whether abrogation of neuronal synchronization would also alleviate pain-like behaviors, mice were microinjected with AAV-hDlx-Gq DREADD-dTomato (Gq DREADD) or the AAV-dDlx-dTomato vector control (Figure 5L and Supplemental Figure 8A). Following FLIT surgery, we used C21 to activate DREADD. Remarkably, the mice that received Gq DREADD exhibited attenuated pain-related behaviors, including spontaneous pain–like behavior, whereas mice that received the vector control displayed spontaneous paroxysmal asymmetrical facial grimaces. Moreover, we also carried out global assessment of pain using composite pain scores, and the results showed that the Gq-DREADD group had significantly lower pain scores (Figure 5, M–O, and Supplemental Figure 8, B–D). As such, activation of S1ULp–S1J GABAergic neurons alleviated pain-like behavior, including spontaneous pain.

### Attenuation of S1 neural dynamics with clinically effective pain treatment.

**Figure 6:**
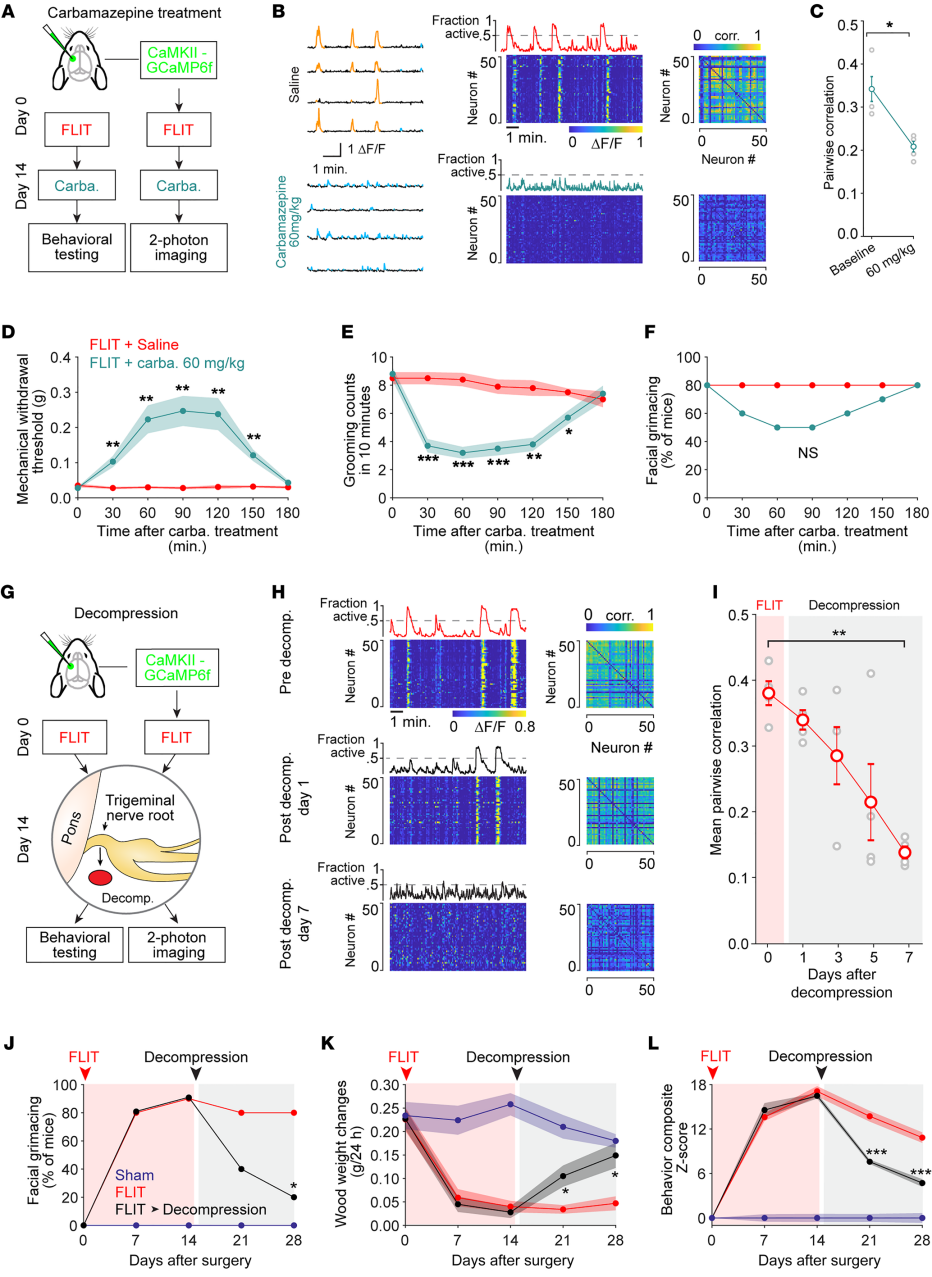
Clinically effective treatments alleviate S1 synchronization and pain-like behavior. (**A**) Diagram depicting the carbamazepine (Carba.) experiment. Excitatory neuron imaging and behavioral testing were carried out on separate groups of animals. (**B** and **C**) Two-photon calcium imaging 14 days after FLIT surgery. Animals (*n* = 4) received normal saline followed by carbamazepine 60 mg/kg with a 12-hour interval. (**B**) Left: Sample calcium transient traces of neurons. Middle: Heatmaps of neuronal activity. Right: Correlation coefficient matrices of the neurons shown in the left panels. (**C**) Pairwise correlation for each treatment for all 4 animals (gray circles). Data indicate the mean ± SEM. *P < 0.05, by paired *t* test. (**D**–**F**) Fourteen days after FLIT surgery, animals received normal saline followed by carbamazepine with a 12-hour interval (*n* = 10). (**D**) Mechanical withdrawal threshold for von Frey filament tests (mean ± SEM). **P < 0.01, by 2-way ANOVA; a post hoc Bonferroni’s test was performed to determine significant differences at the indicated time points for saline versus carbamazepine treatment. (**E**) Facial grooming counts. *P < 0.05, **P < 0.01, and ***P < 0.001, by 2-way ANOVA; a post hoc Bonferroni’s test was performed to determine the *P* value for saline versus carbamazepine treatment. (**F**) Percentage of mice with asymmetrical facial grimacing. No statistical significance (NS) was found between the groups (Fisher’s exact test). (**G**) Flowchart of the experiment for S1 calcium imaging (*n* = 4) and behavioral tests (*n* = 10 per group) for decompression (removal of Surgifoam) of the trigeminal nerve root in FLIT mice. (**H**) Representative neuronal activity heatmaps and correlation coefficient matrices. (**I**) Pairwise correlation coefficient before and after decompression (individual animals are indicated in gray and the mean ± SEM in red). **P < 0.01, by 1-way ANOVA, with a Tukey-Kramer comparison. (**J**–**L**) Behavioral testing of mice subjected to decompression 14 days after FLIT surgery. (**J**) Asymmetrical facial grimacing. *P < 0.05, by Fisher’s exact test. (**K**) Wood-chewing assay. *P < 0.05, by 2-way ANOVA; a post hoc Bonferroni’s test was performed to determine the *P* value for FLIT versus FLIT plus decompression. (**L**) Composite *z* scores for behaviors were computed for all groups. ***P < 0.001, by 2-way ANOVA; a post hoc Bonferroni’s test was performed to determine the *P* value for FLIT versus FLIT plus decompression.

We next asked whether clinically effective pain treatment would attenuate S1 synchrony. Carbamazepine, an antiseizure medication, is currently used clinically as a first-line treatment for TN (35). We therefore administered it to the FLIT model mice (Figure 6A). We found that S1 synchronization was significantly decreased by carbamazepine (Figure 6, B and C). Carbamazepine also dampened mechanical allodynia, reduced the frequency of facial grooming, and modestly reduced facial grimaces, albeit not statistically significantly (Figure 6, D–F), consistent with clinical reports of partial pain resolution with carbamazepine. In contrast, ketorolac, a nonsteroidal antiinflammatory medication with no clinical efficacy against TN (36), did not significantly alter neuronal activity or alleviate pain-like behavior (Supplemental Figure 9, A–I).

We tested whether trigeminal nerve root decompression surgery (Figure 6G), a definitive treatment for patients with TN with vascular compression (37), altered cortical S1 activity dynamics. Population dynamics in S1 showed strongly attenuated synchrony after decompression. By day 7 after decompression, S1 neuronal activity returned to levels similar to pre-FLIT baseline levels (Figure 6, H and I). The decompressed FLIT mice exhibited attenuated mechanical allodynia: the mechanical withdrawal threshold returned to pre-FLIT baselines within 14 days of decompression (Supplemental Figure 10A). Consistent with this, asymmetrical facial grimaces, decreased wood gnawing (Figure 6, J and K), facial grooming, overgrowth of incisors, and body weight phenotypes were also reversed (Supplemental Figure 10, B–D). The composite *z* score derived from pain-related behaviors revealed that decompression reversed the behavioral phenotypes within 14 days (Figure 6L). We also performed a recompression experiment in decompressed mice that had resolved cortical synchrony (Supplemental Figure 10, E and F) and observed a reoccurrence of synchronized S1 activities following recompression, confirming the causal link between induction of pain and synchronized S1 activity.

## Discussion

We showed a dramatic increase in synchronicity of S1 pyramidal neurons as a result of neuropathic pain (Figure 2 and Supplemental Figure 5). This hypersynchrony is not a mere by-product of pain, rather, it is critical for pain-like behavior, including spontaneous pain. The interrogation of cortical mechanisms responsible for spontaneous pain was greatly facilitated by our clinically relevant model of TN. We developed this model after reasoning that cortical neuronal dynamics could be well captured in a model of orofacial pain with robust spontaneous pain–like behavior. The orofacial area occupies a disproportionally large region on the S1 homunculus compared with other body parts (20, 21, 38), enabling the investigation of cortical neural dynamics of pain.

Pathological insults to the nervous system can induce characteristic hypersynchronous states, as reported for Alzheimer’s disease, Parkinson’s disease, and epileptic seizures (39–42). A mechanistic understanding of aberrant neural synchronization in these pathological conditions is currently lacking. We showed that S1 interneuron hypoactivity is key for excitatory neuron hypersynchrony. Selectively activating interneurons in the affected areas reversed hypersynchrony of excitatory neurons and alleviated pain-like behavior, enabling us to gain insights into the mechanisms underlying the observed pathological activity in pain. Such S1 interneuron hypoactivity has been recently shown to mediate sensory hypersensitivity in a mouse model of autism spectrum disorder (43) and may indeed underly a range of other pathologies of the brain. Cortical interneurons have several subgroups, including parvalbumin-expressing, somatostatin-expressing, and vasoactive intestinal peptide–expressing interneurons. Somatostatin-expressing cortical interneurons have been shown to be implicated in neuropathic pain (19). How interneuron subgroups singly or jointly affect cortical excitatory neuron hypersynchrony and pain-like behavior in the FLIT model is unknown.

Cortical hypersynchrony observed in neuropathic pain was functionally important for pain-like behavior. Reversing the S1 hypersynchrony through local interneuron activation ameliorated pain-like behavior (Figure 5), indicating that targeting aberrant pain-induced S1 neural dynamics could relieve pain. Interestingly, antiseizure medication, carbamazepine, which is used as a first-line medication for TN, but not ketorolac, a nonsteroidal antiinflammatory drug, could alleviate S1 hypersynchrony. Consistent with this, trigeminal nerve root decompression, a definitive treatment of TN for a subset of patients with surgically amenable nerve root compression, also attenuated S1 hypersynchrony (Figure 6).

Elevated synchrony of neurons is not necessarily a sign of pathology. Neuronal synchronization in the visual cortex has been shown to establish relations in different parts of the visual field coding for global features of stimuli such as continuity, similarity of orientation, and coherency of motion (44). The olfactory system also demonstrates transiently synchronized neuronal activities in odor-evoked dynamic ensembles. This temporal synchronization is linked to combinatorial representations of time and space during an odor response (45, 46). More recently, synchronization of the cortical layer 5 pyramidal neurons has been linked to loss of consciousness during general anesthesia (47). It is therefore important to delineate pathological synchrony from otherwise healthy cortical function. The pain model we developed provides a reliable method to reversibly induce pathological hypersynchrony. Most existing pain models derive from original insults that could not be reversed in a temporally controlled fashion. As shown (Figure 6 and Supplemental Figure 10), trigeminal nerve root decompression alleviated cortical hypersynchrony, and nerve recompression reintroduced the hypersynchrony. As such, decompression and recompression of the FLIT model changed cortical dynamics in a “predictable” fashion. Our approach, thus, established a robust and reproducible method for investigating how pain can alter cortical microcircuitry linked to intractable, persistent maladaptive behavior.

Recent research has started to investigate the cortical representation of pain. Formalin-induced pain has been found to be associated with neuronal oscillation in the S1 region (48). In a mouse spared-nerve injury model of neuropathic pain, heightened S1 neuronal activity has been shown to mediate pain perception (19). However, in these studies, whether or not cortical neurons exhibit synchrony is unclear. Synchronized cluster firing has been reported in the anesthetized condition in the dorsal root ganglion primary sensory neurons (49). Our results indicate that, in the awake state with no experimental stimuli, localized S1 synchronization is a key cortical pattern mediating pain-like behavior. Connecting these observations yields a new hypothesis that peripheral and central mechanisms orchestrate the neurological manifestations of spontaneous pain.

The use of immediate early genes, particularly c-*Fos*, to map neural activity associated with biological and behavioral perturbations has led to many exciting discoveries (28), including the discovery of anesthesia-related analgesic effects mediated by a group of neurons in the amygdala (50). We leveraged c-*Fos* immunostaining to map pain-related cortical activity in S1ULp–S1J in mice that underwent induction of neuropathic pain. Notably, these mice did not receive any experimentally imposed stimulation to evoke pain responses. Therefore, c-*Fos* expression attributed to internal processing of pain could be derived by comparing animals that underwent FLIT surgery with those that underwent sham surgery. Consistent with c-*Fos* expression, neural activity was significantly increased in S1ULp–S1J neurons (Figure 2 and Supplemental Figure 5). Using TRAP2 mice (28), we interrogated the function of spontaneous pain–related c-*Fos*–expressing neurons and found that these neurons were indispensable for mediating spontaneous pain–like behavior. Combining behavioral and imaging approaches, we observed a regional difference between S1ULp–S1J and S1BF in mediating spontaneous pain (Figure 4 and Supplemental Figure 6). S1BF has been long recognized as a critical region decoding whisker stimulation (51), and our results indicated that cortical processing of spontaneous pain in the orofacial areas involves different mechanisms than vibrissal sensory processing by S1BF. Recently, the dysgranular region was implicated in S1 processing of pain (52). In our orofacial spontaneous pain model, we did not observe significant c-*Fos* expression in this region, which might be related to different types of pain models as well as the presence or absence of spontaneous pain (52).

Taken together, we found that distinct states of S1 hypersynchrony dynamics acted as a key neural substrate for the mediation of spontaneous pain. Our findings provide mechanistic insights into this devastating aspect of human neuropathic pain and open up new avenues for new treatments targeting pathological neural synchrony.

## Methods

### Animal.

Male *Fos^2A-iCreER^* mice (TRAP2) (Jax 030323) and adult male and female C57/BL6 mice (16–24 weeks old) were purchased from The Jackson Laboratory. Mice were housed in a temperature-controlled vivarium on a 12-hour light/12-hour dark cycle (lights on at 0700 hours; lights off at 1900 hours) with ad libitum access to food and water. CD1 male mice (16–26 weeks old,) and male SD rats (10–14 weeks old) were also purchased from The Jackson Laboratory and Charles River Laboratories, respectively. For carbamazepine, oral gavage of carbamazepine (Novartis, packaged by Precision Dose) at 60 mg/kg was used. For ketorolac (Hospira), oral gavage of 10 mg/kg was used. For tamoxifen (MilliporeSigma, WXBD4583V), intraperitoneal injection of 150 mg/kg dissolved in corn oil was used.

### FLIT procedure.

Mice were anesthetized with isoflurane inhalation. Surgery was performed under an Omano surgical microscope (OM2300S-V7, 7-40X). A 1–1.5 cm midline neck incision was used. The superficial tissues were bluntly dissected and lateralized with a mini retractor. The neck muscles were gently dissected to locate the mouse’s right auditory bulla and the auditory capsule on the right side of the head, which are the landmarks to locate the foramen lacerum. A prepared piece of Surgifoam (Ethicon) at approximately 1–1.5 mg was gently delivered into the foramen lacerum using curved forceps. The Surgifoam was positioned between the trigeminal nerve root and the cochlea bulla. After removing the retractor and replacing the tissues, the skin was closed with 6-0 nylon monofilament (Ethicon) sutures. Mice in the sham group underwent the same surgical procedure including neck shaving, skin incision, muscle dissection, and foramen lacerum exposure without nerve root compression. The duration of the surgery ranged from 8 to 12 minutes per mouse. Tamoxifen was intraperitoneally administrated to *Fos*-iCre-ERT2 (*Fos^2A-iCreER^*-knockin) mice immediately after the FLIT procedure.

### Trigeminal nerve root decompression and recompression.

On day 14 after the FLIT procedure, mice were anesthetized with isoflurane in oxygen. The same neck incision and neck cervical dissection were performed as described for the FLIT procedure. After locating the foramen lacerum, the Surgifoam was removed with care. The incision was closed as described in the FLIT procedure. Recompression surgery was performed as described in the FLIT procedure.

### Mechanical withdrawal threshold.

Mice were individually placed in a custom-made box (6 × 6 × 6 cm) with the top, bottom, and 4 walls made of metal mesh and were allowed free movement. After 30 minutes of acclimation, a graded series of von Frey filaments were inserted through the mesh walls from the lateral side and applied to the skin of the vibrissa pad within the trigeminal nerve V2 branch–innervated territory for 1 second at 10-second intervals. A brisk withdrawal of the head upon stimulation was considered a positive response. Mice were tested 5 times, with at least 3 positive responses indicating a positive result. The minimum force necessary to elicit a response was defined as the mechanical withdrawal threshold.

### Observation of face grooming and grimacing.

For the facial grooming and grimacing test, each mouse was habituated for 30 minutes daily for up to 3 consecutive days in a 10 × 10 × 12 cm Plexiglass box equipped with a mirror to record unobstructed views of the orofacial area. The mouse behaviors were recorded for 10 minutes without any extra audio or physical disturbance. Grooming was defined as face-washing strokes primarily directed to the trigeminal nerve impingement side. Facial grimacing for this study was defined as asymmetrical eyelid contraction of the ipsilateral eye (same side as the trigeminal nerve compression) compared with the contralateral side, as determined by blinded observers. The recorded behaviors were analyzed by an experimenter who was blinded to the procedure and group assignments of the mice.

### Food preference.

Mice were deprived of food 12 hours prior to the test, with water accessible ad libitum. To prepare the soft chow, regular solid chow was soaked in water (pellets: water = 1: ~2 g) for 20 minutes. Regular solid chow and freshly prepared soft chow were placed on plates. The test mouse was videotaped using a camera 40 cm above the cage for 10 minutes. The time spent eating solid and soft chow in each video was quantified by experimenters who were blinded to group assignments.

### Wood-chewing assay.

Balsa wood blocks were custom-sized to 1 inch cubes. Mice were housed in individual cages with food and water supplied ad libitum, and a wood block was placed in the cage for 24 hours. The weight of the block before and after placement in the cage was recorded.

### Behavioral composite z score.

For a comprehensive assessment of several pain-related parameters, we used the following formula: *z*  = [Δ *X*Surgery − Mean(Δ *X*)Sham]/SD (Δ *X*)Sham (53). In the formula, Δ *X*Surgery was the score for the mice in the surgery group at different time points minus the score for these mice at day 0 baseline; Mean(Δ *X*)Sham was the score for mice in the sham group at different time points minus the score for these mice at day 0 baseline; and S(Δ *X*)Sham was the SD of Δ *X*Sham for any given time point. Specifically, the composite *z* score for the mouse was calculated as the sum of the 6 *z* score values (mechanical withdrawal thresholds, grooming counts, body weight, wood chewing, incisor length, and food preference) normalized to the SD for that sum for the sham controls.

### Mouse sexual function.

For sexual function, naive female mice were used as mating partners. Male mice were placed in a cage with a camera positioned 40 cm above to record mating behavior. The mounting time and attempts were obtained from the videos by experimenters who were blinded to the study design.

### Testosterone quantification.

Urine testosterone quantification was performed according to the manufacturer’s recommendation (R&D Systems, catalog KGE010). Briefly, the assay plate was prepared with primary antibodies at room temperature for 1 hour. Urine samples were added to each well and incubated for 3 hours at room temperature, followed by substrate reaction at room temperature for 30 minutes. A microplate reader (450 nm) was used to determine the optical density of each well.

### Open-field test.

Mice were habituated for 30 minutes before the test to allow acclimation to the testing environment. Each mouse was placed in a 40 × 40 cm wall-enclosed box, with concurrent activation of the SMART video tracking system (SMART software vision 3.0, Panlab, now Harvard Apparatus), and locomotion activity was recorded for 10 minutes with minimal external stimuli. Behavior parameters, including the percentage of time spent in the center zone and the latency to the first entry into the center zone were automatically tabulated by the software and analyzed by an experimenter who was blinded to the study design.

### Conditioned place preference.

Conditioned place preference (CPP) testing was performed as previously described (54). Briefly, CPP was performed in a 3-chamber apparatus (Med-Associates) containing a white and a black compartment (20.3 × 15.9 × 21.3 cm) with distinct patterns on the floors, separated by a central gray neutral area. Male *Fos^2A-iCreER^* mice were injected with either AAV8-hSyn-DIO-hM4D(Gi)-mCherry (Addgene 44362) or vector virus AAV8-hSyn-DIO-mCherry (Addgene 50459) at day –28. At day 0, these animals received FLIT or the sham procedure, immediately followed by a single intraperitoneal injection of tamoxifen (150 mg/kg). On day 7 after FLIT surgery, the animals were screened using a preconditioning test. During the preconditioning test, mice were allowed 10 minutes of free access to all compartments. Mice that spent less than 75% of their time in any 1 compartment were included in the study. The conditioning phase started on day 8 after FLIT surgery. During the conditioning phase, mice were confined to 1 compartment for 45 minutes after an intraperitoneal injection of C21 (1 mg/kg), or to the other compartment after a saline injection with a 6-hour interval. After 7 consecutive days of conditioning, mice were retested. The percentage of time spent in the paired compartment was calculated for each mouse as T2/(T1+T2) × 100, where T1 and T2 represent the time spent in the unpaired and paired compartments, respectively. The CPP score was calculated for each mouse as [(W2−W1)/W1] × 100, where W2 represents the percentage of time spent in the C21 paired compartment during the final test and W1 represents the percentage of time spent in the same compartment during the initial test.

### Botulinum toxin A injection.

Mice were briefly anesthetized under isoflurane anesthesia. To be consistent with the FLIT procedure on the right side, masseter muscles on the right side were injected with botulinum toxin A (Merz Pharmaceutical) at 0.4U in 50 μL. H&E staining atrophy was diagnosed by a pathologist who was blinded to group assignments, using muscle fiber diameter and nucleus position as the diagnostic criteria.

### Craniotomy and virus injection.

Cranial windows were implanted into the contralateral side for the FLIT and sham procedures. Mice were anesthetized with isoflurane. The eyes were moistened with eye lubricant. To minimize postoperative pain, ketorolac tromethamine (Althenex) was administrated (5 mg/kg) intraperitoneally every 24 hours for 3 consecutive days. The fur on the top of the head was shaved between the outer canthus and concha, and then the mouse was positioned in a stereotactic frame with a head holder. The skin was prepared with povidone-iodine solution (Aplicare) followed by a 70% alcohol swab (BD). After lidocaine (0.2 mL, 1%) infiltration, a skin flap overlying the dorsal skull was removed using microscissors. The connective tissues and periosteum of the parietal skull were thoroughly cleaned. A 3 × 3 mm piece of bone was removed to reveal the left anterolateral cortex including the S1BF, S1ULp, and S1J regions as determined by stereotactic coordinates following Chen et al. (43), and the dura was kept moist with sterile saline.

For GCaMP6f expression in pyramidal neurons of the targeted cortex, adeno-associated virus 8 (AAV8) carrying CaMKII-GCaMP6f (pENN.AAV.CamKII.GCaMP6f.WPRE.SV40; Addgene 100834; 1 × 10^12^ genome copies/mL) was injected with the Nanoject III (Drummond Scientific Company, model 3-000-207) at a depth of 200 μm beneath the pia surface, and virus was slowly injected into 4–5 sites approximately 1–4 mm lateral to the midline of the skull and bregma approximately 1 to –2 mm in WT mice. Cre-dependent GCaMP6f virus (AAV.Syn.Flex.GGaMP6f.WPRE.SV40, Addgene 100833, 1 × 10^12^ genome copies/mL) was selectively injected within the S1J (jaw) and S1ULp (upper lip) cortex coordinated at 0.5–1.5 mm to bregma, approximately 2.8–3.5 mm lateral to the midline in *Fos*^2A-iCre^ TRAP2 mice; for the S1BF cortex injection, Cre-dependent GCaMP6f virus was injected at bregma –0.5 to –1.8 mm, and 3–3.5 mm lateral to the midline in *Fos*^2A-iCre^ TRAP2 mice. For expression of GCaMP6f in GABAergic neurons, GCaMP6f in AAV8 under the Dlx5/6 promoter AAV (pAAV-mDlx-GCaMP6f-Fishell-2; plasmid 83899) was injected into the same area of the S1ULp (upper lip) and S1J (jaw) of WT mice.

For expression of DREADDs in GABAergic or glutamatergic neurons, pAAV9-hDlx-GqDREADD-dTomato (Addgene plasmid 83897) or AAV8-hSyn-DIO-GiDREADD-mCherry (Addgene plasmid 44362) was injected into the S1ULp and S1J at approximately 2.8–3.5 mm lateral to the midline, bregma 1.5 to –0.5 mm. For S1BF injection, AAV8-hSyn-DIO-GiDREADD-mCherry was injected at bregma –0.5 to –1.8 mm, and 3–3.5 mm lateral to the midline in TRAP2 mice. Control mice underwent the same procedure, with injection of the same volume of pAAV9-hDlx-dTomato or AAV-hSyn-DIO-mCherry (vector control) into the S1ULp and S1J regions, respectively.

For cranial window implantation, 2 circular, presanitized glass coverslips, 3 mm and 5 mm (Warner Instruments, nos. 64-0700 and 64-0720) in diameter, were individually conjoined with optical adhesive (Norland Products, no. 417). The 3 mm coverslip was laid over the pia surface within the craniotomy, and the surrounding skull was covered by the 5 mm coverslip. A custom-designed head plate was adhered over the glass window with adhesive luting cement (C&B Metabond, 171032, Parkell).

For wide-field imaging of calcium dynamics, AAV-CaMKII-GCaMP6f was injected across a large area of the cortex covering S1ULp, S1J, S1 barrel cortex, S1 forelimb, S1 hind limb, M1, M2, S2, etc. (midline to 3 mm at bregma ~1.5 mm and to ~4 mm at bregma –2 mm). Four weeks were allowed for virus expression. For the wide-field brain imaging window, a trapezoidal craniotomy was done from the midline to 3 mm at bregma ~2 mm and to ~4.85 mm at bregma –3.5 mm, respectively, lateral to the midline. The brain cortex was covered by a customized glass coverslip, and a metal headplate was attached using adhesive luting cement.

### Two-photon imaging.

Prior to the imaging session, mice were taken to the two-photon microscope room and placed on a microscope stage using a head fixation device for 30 minutes per day over 5–7 days. In vivo two-photon imaging was performed with a two-photon system (Ultima, Bruker) equipped with a Mai Tai Laser (Spectra Physics, KMC 100). The laser was tuned to 910 nm, and the average laser power through the transcranial window was approximately 20–30 mW for both excitatory neuron and interneuron image acquisition using a ×20, 1.0 NA water-immersion objective (Olympus). A regular ×4 objective (Olympus) was used for wide-field viewing of the S1 cortex. All images except for those acquired in the pilocarpine experiment were acquired at a frame rate of 6–12 Hz for approximately 15 minutes using Prairie View Software in awake, nonanesthetized mice. A 3 Hz frame rate was used for wide-field S1 cortex imaging with a ×4 objective. For mice that received pilocarpine, images were acquired for approximately 3 minutes instead of 15 minutes to minimize restraint of the animals during epilepsy. Of note, for any given animal, the field of view was kept constant to obtain images of the same group of neurons longitudinally.

### Calcium imaging data analysis.

Imaging data were corrected for motion between frames using the NoRMCorre software package (55). Neuron selection was carried out subsequently using custom-written software in MATLAB (MathWorks). Calcium fluorescence signals of each individual neuron were extracted from the corrected video files. The signal for each neuron was corrected for background fluorescence changes by subtracting the fluorescence changes from the immediate surrounding. Each neuron’s activity time course was then quantified using the formula Δ*F* = (*F* – *F0*)/*F0*, where *F* is the fluorescence signal at a given frame, and *F*0 was calculated from a sliding window of ±30 seconds around the frame. Finally, baseline correction was carried out by fitting a linear function (MATLAB function robustfit) to the low-pass filtered (cutoff: 0.3 Hz) signal. A deconvolution algorithm (Fast online deconvolution of calcium imaging data) was applied to detect transients (56). The start and end of transients were detected when the model was above 0.1.

Global events were detected when the fraction of simultaneously active neurons exceeded 50% of all neurons in that recording in a given frame. An active neuron refers to a neuron exhibiting a transient that was detected at that frame. Subsequently, a transient was categorized as “global” if any part of it overlapped with a global event. Otherwise, a transient was categorized as independent. The fraction of global transients was calculated by dividing the number of global transients by the total number of transients of a given neuron. Only neurons with at least 5 detected transients in a given recording were included in this analysis. The fraction of global transients per neuron was calculated in the same way as previously described, with normalization for each recording. A second-order polynomial function was used to fit to the neurons of each animal.

Pairwise correlation analysis was carried out by calculating the Spearman’s rank correlation coefficient for each pair of neurons in a recording. For each correlation matrix, the autocorrelation (i.e., the correlation of a neuron with itself) was not included in any analysis and is shown in dark blue in the correlation matrices. The average correlation values were calculated as the mean of all pairwise correlations in a recording.

Network analysis was carried out using principal component analysis (PCA) (MATLAB function PCA using SVD) and retaining only the first 3 principal components. Periods in which a global event was detected are highlighted in gold in Figure 2H, Figure 3D, and Supplemental Figure 5C. The distance of the network trajectory from the mean was evaluated by taking the mean value for each coefficient and calculating the Euclidean distance of each time point to the mean. This was done for time points that belonged to a global event and those that did not.

Wide-field calcium imaging using a ×4 lens was analyzed by extracting signals of each pixel in the imaging field, and the Δ*F/F* for each frame was calculated for each pixel without drawing the region of interest. The heatmap was generated by averaging the Δ*F/F* for each pixel over time.

Total integrated calcium activity was quantified by calculating the mean total AUC for all neurons in a recording. The AUC was normalized for each animal, and the relative change across days was calculated as the mean across animals.

### c-Fos staining.

Mice were briefly maintained under isoflurane anesthesia, and procedures were performed after lidocaine 1% infiltration of the incision sites. After a 2-hour recovery period, the mice were sacrificed and immediately perfused with ice-cold PBS followed by 4% paraformaldehyde (PFA) in 0.1 M phosphate buffer (4% PFA). Brain samples were fixed in 4% PFA at 4°C for 24 hours. Coronal brain sections were sliced at 60 μm thickness using a Leica vibratome (VT 1000s). Slices from bregma 1.6 mm to –1.8 mm were obtained to cover the anterolateral S1 region including S1J, S1ULp, and S1BF cortex regions. Ten slices covering anterolateral S1J (bregma 1.6 to 1 mm), 15 slices covering S1ULp (bregma 1 to 0 mm), and 15 slices covering S1BF (bregma –0.8 to –1.8 mm) were used for c-*Fos* combined with VGLUT1, VGLUT2, or GAD67 staining.

For double-staining, slices were washed 3 times in PBS for 5 minutes, followed by blocking with 6% goat serum and 2% BSA in PBS with 0.3% Triton X-100 (blocking solution) at room temperature for 1 hour. Floating slices were stained with a primary antibody (rabbit anti–c-*Fos*, Cell Signaling Technology, catalog 2250, 1:500 dilution; anti-VGLUT1, Invitrogen, Thermo Fisher Scientific, catalog 48-240-0, 1:500 dilution; guinea pig anti-VGLUT2, MilliporeSigma, catalog AB2251-I, 1:500 dilution; anti-GAD67, MilliporeSigma, catalog MAB5406) in blocking solution at 4°C for overnight. After washing 3 times with PBS for 5 minutes, slices were incubated with a secondary antibody (goat anti–rabbit Alexa Fluor 488, Jackson ImmunoResearch, catalog 111-545-144, 1:2,000 dilution; donkey anti–mouse Cy3, Jackson ImmunoResearch, catalog 715-165-150, 1:2,000 dilution). Images were acquired with a Nikon A1 confocal microscope equipped with ×20 and ×4 objectives. The acquired images were analyzed with ImageJ software (NIH). The number of positive cells was counted by an experimenter who was blinded to group assignments. For each group, 4 animals were used. The number of c-*Fos*^+^, GLUT1^+^, and GAD6^+^ cells was counted within the slices covering the S1J, S1ULp, and S1BF regions individually.

### DREADD expression and GCaMP staining.

For the expression of DREADDs in GABAergic or glutamatergic neurons, pAAV9-hDlx-GqDREADD-dTomato (Addgene plasmid 83897) or AAV8-hSyn-DIO-GiDREADD-mCherry (Addgene plasmid 44362) was injected into the S1ULp and S1J regions at approximately 2.8–3.5 mm lateral to the midline, bregma 1.5 to –0.5 mm. For S1BF injection in TRAP2 mice, AAV8-hSyn-DIO-Gi-DREADD-mCherry was injected at bregma –0.5 to –1.8 mm and 3–3.5 mm lateral to the midline. To determine DREADD expression, mice were sacrificed and immediately perfused with ice-cold PBS followed by 4% PFA in 0.1 M phosphate buffer (4% PFA). Brain samples were fixed in 4% PFA at 4°C for 24 hours. Coronal brain sections were sliced at 50–70 μm thickness using a Leica vibratome (VT 1000s). The dTomato or mCherry expression site was examined by imaging the coronal sections from bregma 1.6 mm to –1.8 mm using a wide-field fluorescence microscope (Olympus), and images were scanned with a standard ×10 objective. For GCaMP staining, slices were washed 3 times in PBS for 5 minutes, followed by blocking with 6% goat serum and 2% BSA in PBS with 0.3% Triton X-100 (blocking solution) at room temperature for 1 hour. Floating slices were stained with a primary antibody (mouse anti-GFP polyclonal, Invitrogen, Thermo Fisher Scientific, catalog A6455, 1:500 dilution) in blocking solution at 4°C overnight. After washing 3 times in PBS for 5 minutes, slices were incubated with a secondary antibody (goat anti–rabbit Alexa Fluor 488, Jackson ImmunoResearch, catalog 111-545-144, 1:2,000 dilution). The expression of DREADDs (dTomato) and GCaMP was examined under a confocal microscope (Nikon) using excitation wavelengths of 554 nm and 488 nm, respectively.

### Gi- and Gq-DREADD activation for behavioral studies.

Intraperitoneal injection of C21 (Tocris, catalog 5548) was performed, and the dosing regimen was determined according to the experimental design.

### Data and code availability.

The two-photon imaging analysis code can be accessed at https://github.com/harnett/Shiqian-analysis (commit ID: 12fa87b). RNA-Seq data have been deposited in the NCBI’s Gene Expression Omnibus (GEO) database (GEO GSE162284).

### Statistics.

All the data are expressed as the mean ± SEM. Based on our previous studies, sufficient power to detect significance required 6–8 mice per group for the behavioral experiments; 4 mice per group for the immunostaining analyses; 3–5 mice per group for the analyses of two-photon imaging; and 4 mice per group for gene sequencing. The difference in pain behaviors was analyzed using a repeated-measures, 2-way ANOVA. Post hoc comparisons with Bonferroni corrections were used for comparisons across groups at the indicated time points. Facial grimacing was compared across groups using a 2-sided Fisher’s exact test. One-way ANOVA followed by a Tukey-Kramer post hoc comparison was used for pairwise correlation coefficient analysis, and a aired *t* test was used for pairwise correlation analysis of two-photon imaging data. Two-tailed, unpaired *t* tests were carried out to determine the difference in GLUT1 versus GAD67 staining. The difference in fraction global transients and the mean pairwise correlation coefficient for two-photon imaging data were compared using a 2-way ANOVA followed by Bonferroni’s post hoc analyses. A *P* value of less than 0.05 was considered statistically significant, and significance testing was 2-tailed in 2-group comparisons. For Bonferroni corrections, the adjusted *P* values, calculated by dividing the *P* values by the sample size, are reported. Statistical analysis was carried out using GraphPad Prism 8.0 (GraphPad Software).

### Study approval.

All animal use and procedures applied according to protocols approved by the IACUC of MGH. The experiments performed were in compliance with the guidelines established by the NIH and the International Association for the Study of Pain.

## Author contributions

WD, LF, QC, MTH, and SS conceptualized the study. WD, LF, QC, ZL, PJ, GF, MTH, and SS designed the study methodology. WD, LF, QC, LY, ZY, KH, XW, XZ, LC, SW, SX, PH, WC, SZ, CB, DD, TMD, CW, and BW performed experiments. WD, LF, QC, ZL, LY, PJ, MTH, JM, and SS conducted formal analysis and curated data. Writing: Original draft: WD, LF, QC, MTH, and SS wrote the original draft of the manuscript. SS acquired funding for the study.

## Supplementary Material

Supplemental data

Supplemental video 1

Supplemental video 2

Supplemental video 3

## Version 1 01/05/2023

In-Press Preview

## Version 2 03/01/2023

Electronic publication
