# Supplementary material for: Highly synchronized cortical circuit dynamics mediate spontaneous pain in mice
Source: J Clin Invest. 2023 Mar 1;133(5):e166408. doi: 10.1172/JCI166408 (PMC9974100; doi:10.1172/JCI166408)
Supplement: Supplemental data [file jci-133-166408-s126.pdf]

Supplementary materials

Supplementary figures 1 to 10

Movies S1 to S3

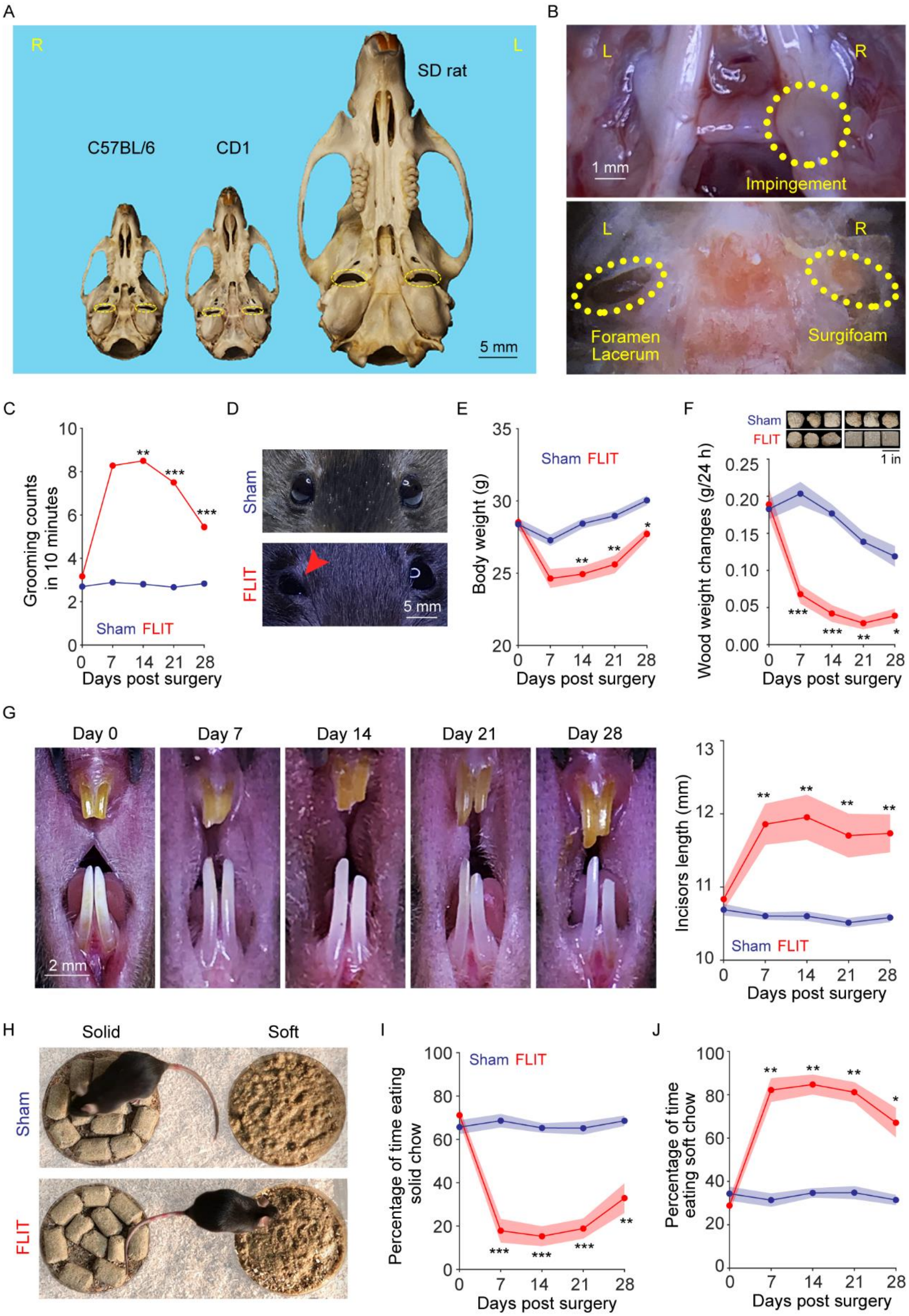

Figure. S1. FLIT model. (A) Skull base of different species with foramen lacerum labeled in yellow ellipses. Scale bar represents 5 mm. **(B)** A representative picture of trigeminal nerve root

impingement from a mouse sacrificed at day 28 post FLIT surgery. Upper panel: top-down view with brain removed to show impingement of trigeminal nerve root on the right side. Lower panel: top-down view with brain removed and trigeminal nerve root lifted to show the Surgiform remained in situ at day 28 post FLIT surgery. Scale bar represents 1 mm. **(C to J)** Behavioral testing for the FLIT model. Mice underwent Sham and FLIT surgery followed by behavioral testing at indicated time points. Equal numbers of male and female mice were used for each group (n = 18, Mean ± SEM.). **(C)** Facial grooming counts (in 10 minutes) at indicated time points. Two-way ANOVA indicates significant difference present between the groups, post-hoc Bonferroni test indicates P values of FLIT *vs.* sham, **$P < 0.01$; ***$P < 0.001$. **(D)** Representative pictures of mouse eyes between groups. Asymmetric eye grimacing was present only in the FLIT group. Scar bar represents 5 mm. **(E)** Body weight of mice were examined at indicated time points. Two-way ANOVA indicates significant difference present between the groups, post-hoc Bonferroni test indicates the P value of FLIT *vs.* IoN-CCI *$P < 0.05$; **$P < 0.01$. **(F)** Top: A representative picture of wood chewing assay at baseline (day 0) and day 7 post surgery. Scale bar represents 1 inch. Bottom: Wood chewing assay to measure wood weight changes for animals that underwent Sham or FLIT surgery. FLIT mice exhibited significant less wood chewing activity compared with sham mice at days 7, 14, 21, and 28. Two-way ANOVA indicates significant difference between the groups, post-hoc Bonferroni test was carried out to determine the P value of FLIT *vs.* sham group, * $P < 0.05$; ** $P < 0.01$; *** $P < 0.001$. **(G)** Left panel: Representative pictures of incisors taken for the same mouse to demonstrate incisors overgrowth in the FLIT model. Scale bar represents 2 mm. Right panel: Quantification of incisors length. Two-way ANOVA indicates significant difference present between the groups, post-hoc Bonferroni test was carried out to determine the P value of FLIT *vs.* sham group, **$P < 0.01$. **(H)** Pictures of food preference assay. **(I)** Percentage of time eating solid chow. FLIT mice exhibited significantly less solid chow eating compared with sham mice at days 7, 14, 21, and 28. **(J)** Percentage of time eating soft chow. FLIT mice exhibited significantly more solid chow eating compared with sham mice at indicated timepoints. Two-way ANOVA followed by post-hoc Bonferroni test was carried out to determine the P value of FLIT *vs.* sham, *$P < 0.05$; **$P < 0.01$, ***$P < 0.001$).

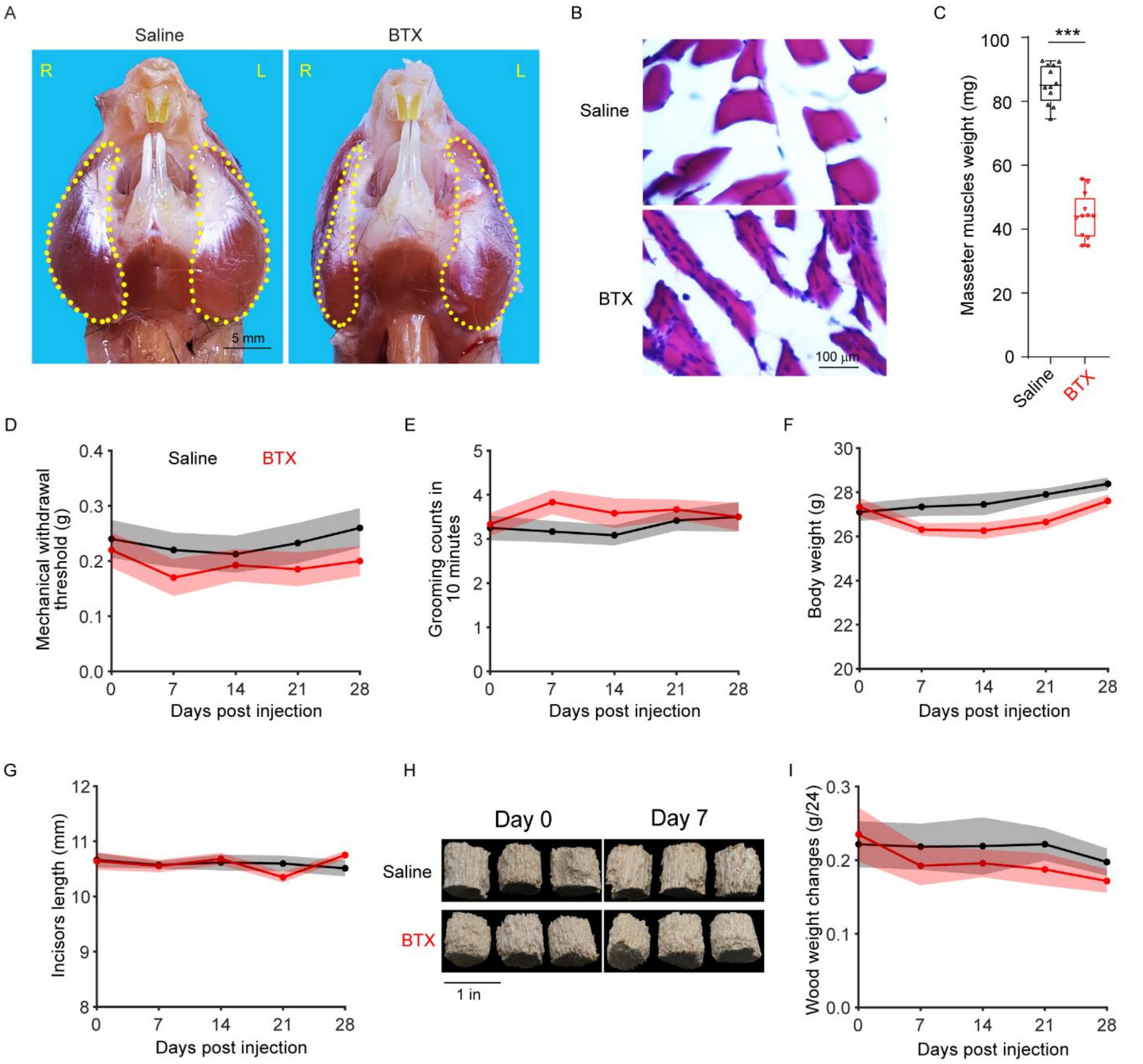

Figure S2. Masseter muscle atrophy does not lead to TN-like behaviors. (A) Representative pictures of mouse masseter muscle 28 days post injection with 50 µl saline (left panel) or 50 µl Botulinum toxin-A (0.4U) (right panel). Both saline injection and Botulinum injection were performed on the right side, consistent with the FLIT procedure. n = 12 per group. Scale bar represents 5 mm. **(B)** Masseter muscle atrophy revealed by H&E staining. Representative H&E staining of masseter muscle shown (40X). Scale bar represents 100 µm. **(C)** Masseter muscles weight was examined at 28 days after injection. Unpaired t test with two-tailed P value indicates significant difference present between the two groups (***$P < 0.001$). **(D-I)** Behavioral testing for the mice received saline or BTX-A injection. n = 12 per group. **(D)** Mechanical withdrawal thresholds to von Frey filaments (mean ± SEM). Two-way ANOVA indicates no significant difference between the two groups. **(E)** Facial grooming counts in 10

minutes at indicated time points. Two-way ANOVA indicates no significant difference between the two groups. **(F)** Body weight of mice received saline or BTX-A injection were examined at indicated time points. Two-way ANOVA test indicates no significant difference between the two groups. **(G)** Incisors length of mice were quantified at indicated time points. Two-way ANOVA test indicates no significant difference between the two groups. **(H)** Representative pictures of chewed balsa wood blocks at indicated time points. **(I)** Quantification of balsa wood weight changes. Two-way ANOVA test indicates no significant difference between the two groups.

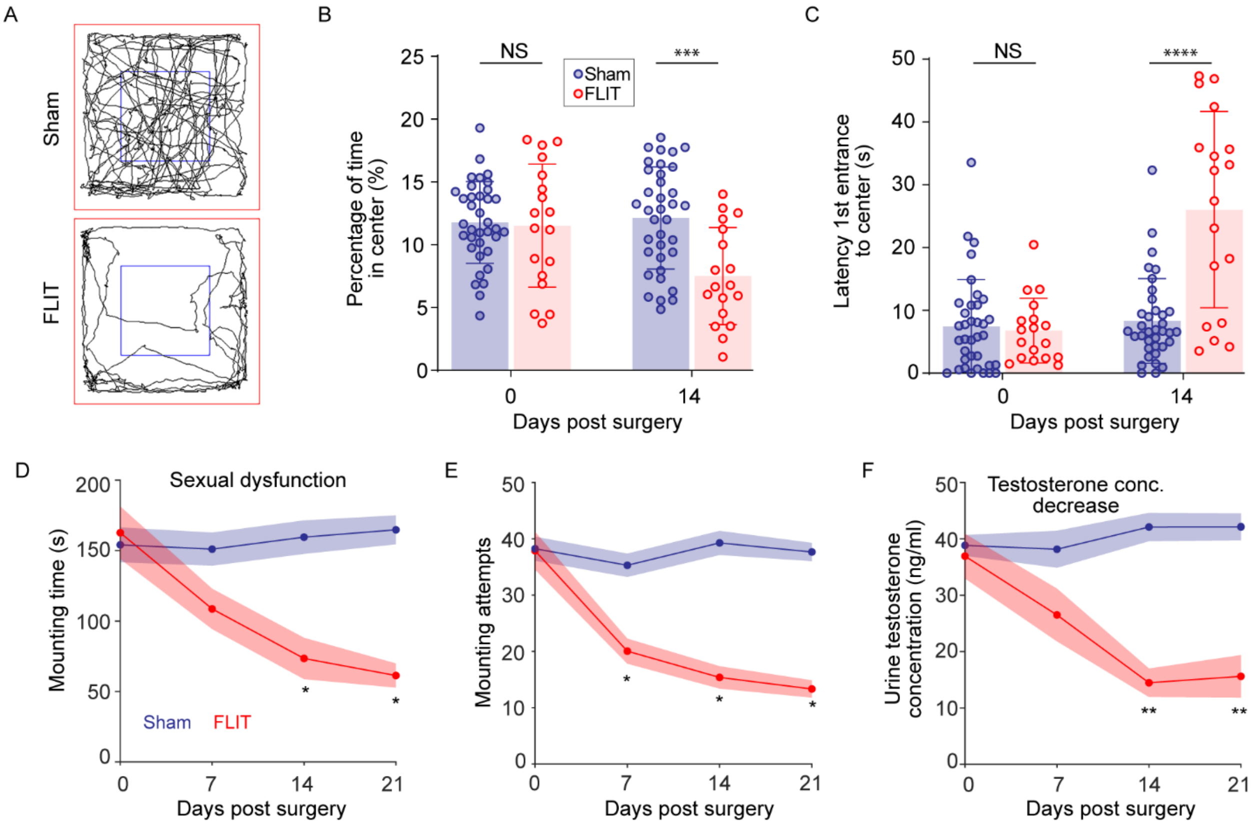

Figure S3. FLIT model develops anxiety-like behaviors and sexual dysfunction. (A to C) Anxiety-like behavior was assessed using open-field assay for mice 14 days post surgery (Sham n = 36, FLIT n = 18, equal numbers of females and males were used). **(A)** Representative trajectories of locomotion. Each tracing represents a travel trajectory of a mouse during a 10-minute testing period. **(B)** Percentage of time spent in central zone. **(C)** Latency of 1st entry into central zone (central zone: blue box in panel (A). Two-way ANOVA followed by post-hoc Bonferroni test was carried out to determine the P value of FLIT *vs.* sham. ***P < 0.001; **** P < 0.0001. **(D to F)** Sexual behaviors test. **(D)** Mounting time was counted for male mice when female partners were present (n = 18 male per group). **(E)** Mounting attempts was counted for male mice when female partners were present (n = 18 male per group). **(F)** Urine testosterone levels of FLIT mice were significantly lower than the sham group at days 14 and 21 post surgery. Urine samples of three mice were pooled together which led to six urine samples per group for all time points. Two-way ANOVA followed by post-hoc Bonferroni test was carried out to determine the P value of FLIT *vs.* sham. *P < 0.05, **P < 0.01.

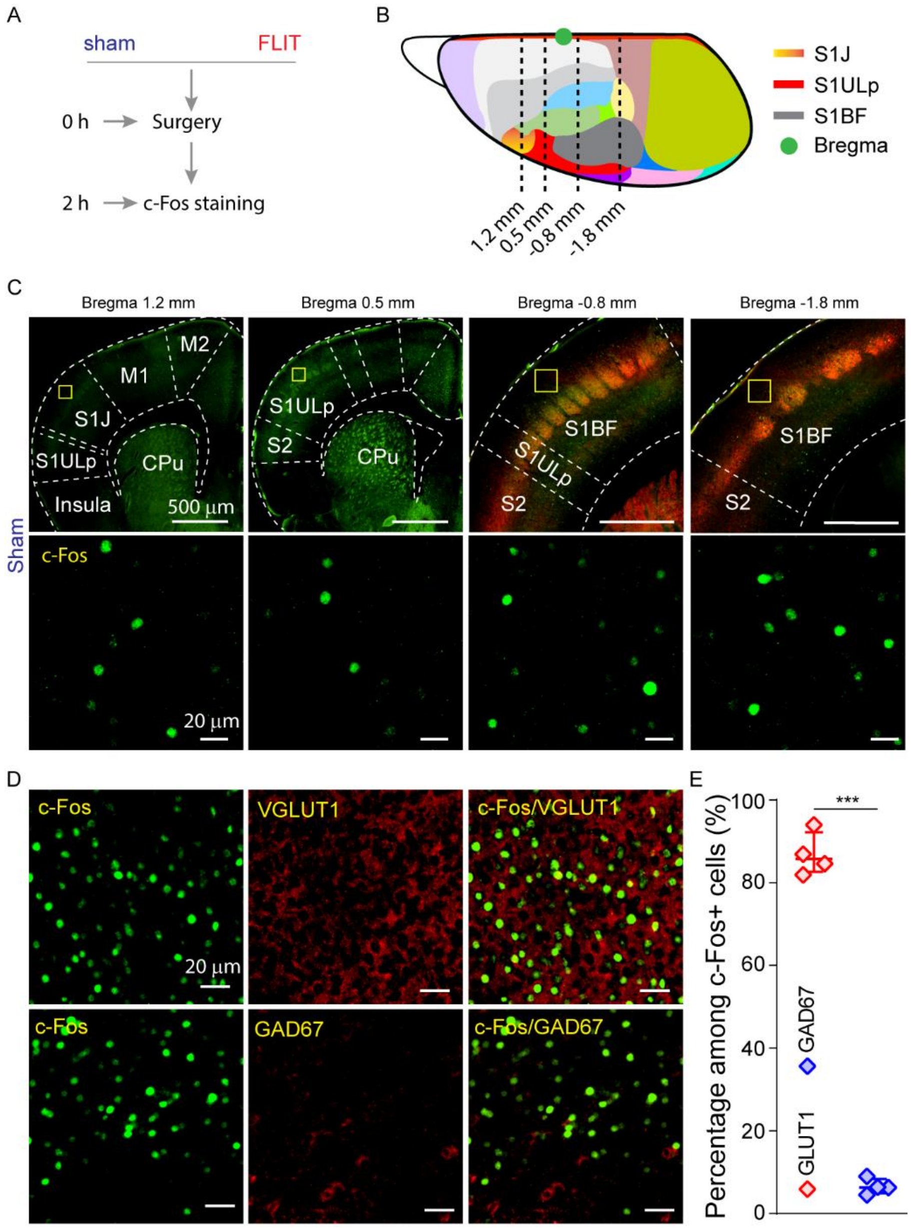

Figure S4. c-*Fos* expression pattern. (A) Diagram depicting c-*Fos* staining to assess neuronal activation after FLIT or sham surgery. **(B)** Schematic of mouse cortex with different colors representing specific cortical regions. Orange represents S1J, red represents S1ULp, and dark grey represents S1BF. Ten slices centered bregma 1.2 mm at 60 μm in thickness were used to assess c-*Fos* expression in S1J; fifteen slices centered bregma 0.5 mm at 60 μm in thickness were used to assess c-*Fos* expression in S1ULp; and fifteen slices between bregma -0.8 mm and -1.8 mm were used to assess c-*Fos* expression in S1BF. **(C)** Representative tangential slices of c-*Fos* staining of sham mice at 4X and 10X. Sequential slices from left to right represent coronal sections covering S1J (bregma 1.2mm), S1ULp (bregma 0.5mm), anterior

S1BF (bregma -0.8mm) and posterior S1BF cortex (bregma -1.8mm). Slices between bregma -0.8 mm and -1.8 mm were co-stained with VGLUT2 (red) to visualize barrels. Lower panels represent boxed regions of corresponding upper panels. **(D)** Representative staining of c-*Fos* and VGLUT1 or GAD67 in S1ULp-S1J of FLIT group. **(E)** Percentage of VGLUT1+ and GAD67+ cells among c-*Fos*+ cells. Two-tailed unpaired t-tests were carried out to determine the difference of GLUT1 *vs.* GAD67. Data are presented as mean ± SEM. *** $P < 0.001$.

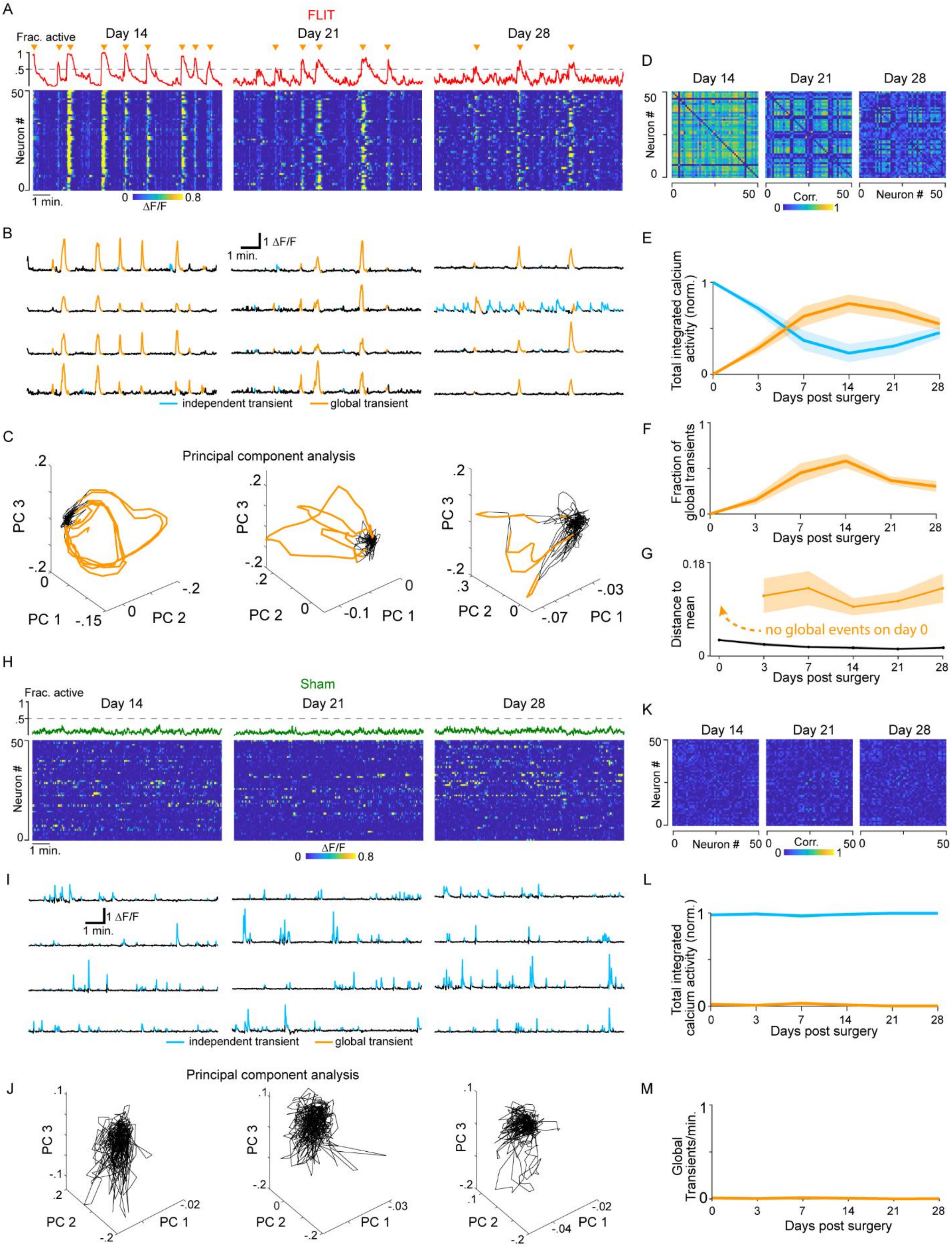
A
FLIT
Frac. active
Day 14
Day 21
Day 28
Neuron #
1 min.
ΔF/F
0
0.8
B
1 ΔF/F
1 min.
independent transient
global transient
C
Principal component analysis
PC 1
PC 2
PC 3
D
Day 14
Day 21
Day 28
Neuron #
Corr.
E
Total integrated calcium activity (norm.)
Days post surgery
F
Fraction of global transients
Days post surgery
G
Distance to mean
no global events on day 0
Days post surgery
H
Sham
Frac. active
Day 14
Day 21
Day 28
Neuron #
1 min.
ΔF/F
I
1 ΔF/F
1 min.
independent transient
global transient
J
Principal component analysis
PC 1
PC 2
PC 3
K
Day 14
Day 21
Day 28
Neuron #
Corr.
L
Total integrated calcium activity (norm.)
Days post surgery
M
Global Transients/min.
Days post surgery

Figure S5. Neural activity on days 14-28. (A) Activity of all recorded neurons on days 14, 21 and 28 of the same FLIT mouse shown in Figure 2C. Fraction of simultaneously active neurons shown above heatmap. Global events are indicated by yellow triangles. **(B)** Four sample traces of calcium transients were shown with independent and global transients indicated. **(C)** Trajectories of neuronal populations plotted as a function of the first three principal components. Episodes highlighted in yellow correspond to global events. **(D)** Pairwise correlation matrices (Spearman's rank correlation coefficient) for the days shown in (A). **(E)** Total integrated calcium activity for global and independent transients calculated as area under the curve (AUC), normalized to the total AUC. **(F)** Frequency of global transients as a function of days post FLIT surgery. Yellow lines indicate values for individual animals. **(G)** Distance of neuronal trajectory during global events to the mean for all FLIT animals. The distance was calculated as the Euclidean distance of the first three principal components from the mean value of those components. Indicated in yellow line is the mean Euclidean distance of datapoints belonging to global events. No global events were detected on day 0. **(H to M)** Same as above for the sham group.

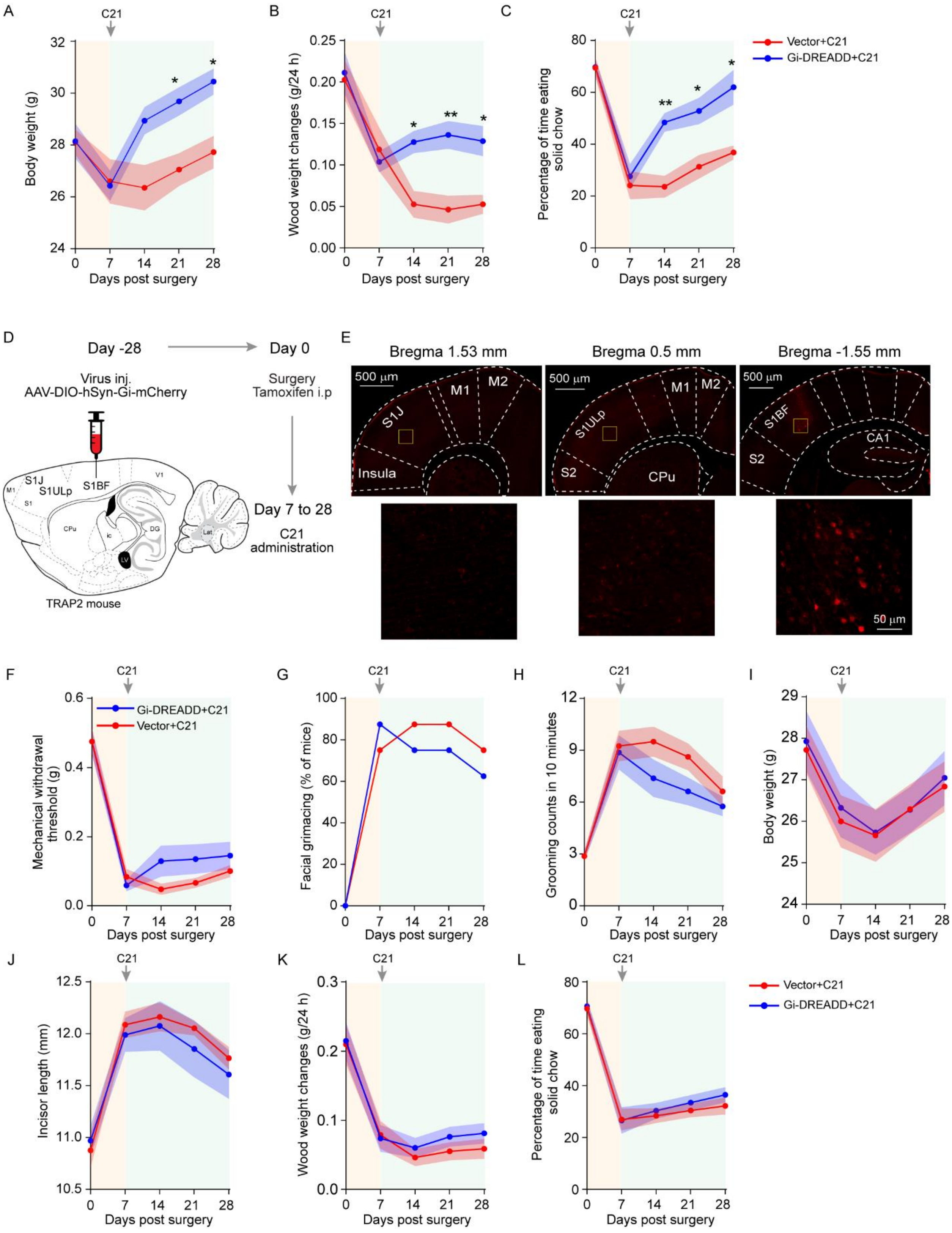

Figure S6. Chemogenetic manipulation of S1ULp-S1J but not S1BF suppresses pain-like behaviors. (A to C) Chemogenetic inhibition of c-*Fos*-induced Gi-expressing neurons leads to

attenuated pain-like behavior. Behavioral testing was performed at indicated timepoints. At day 7, behavioral testing was performed prior to C21 administration to obtain pre-treatment baseline. **(A)** Body weight. **(B)** Wood weight changes in 24 hours. **(C)** percentage of time eating solid chow. Two-way ANOVA followed by Bonferroni post hoc test was carried out to determine the difference between the two groups. Data are presented as mean ± SEM. * $P < 0.05$, ** $P < 0.01$. **(D to L)** TRAP2 mouse was injected with AAV-DIO-hSyn-Gi-mCherry or AAV-DIO-mCherry vector in S1BF at day -28 ($n = 8$ per group). FLIT surgery was performed for all mice at day 0 accompanied by tamoxifen administration. From day 7, C21 was intraperitoneally administrated twice daily. Mice were sacrificed at day 28 for brain slices. **(D)** Diagram of virus injection and flowchart of experiment timeline. **(E)** Upper panels: Representative tangential slices of S1J, S1ULp and S1BF demonstrating mCherry expression was primarily located in S1BF. Lower panels represent boxed regions of corresponding upper panels. **(F)** mechanical withdrawal threshold to von Frey filaments. **(G)** Percentage of mice with facial grimacing. **(H)** Face grooming counts in 10 minutes. **(I)** Body weight. **(J)** Incisors length. **(K)** Wood weight changes in 24 hours. **(L)** Percentage of time eating solid chow. Two-way ANOVA followed by Bonferroni post hoc test was carried out to determine the difference between the two groups. Data are presented as mean ± SEM. Fisher's exact test was used to determine statistical difference for facial grimaces.

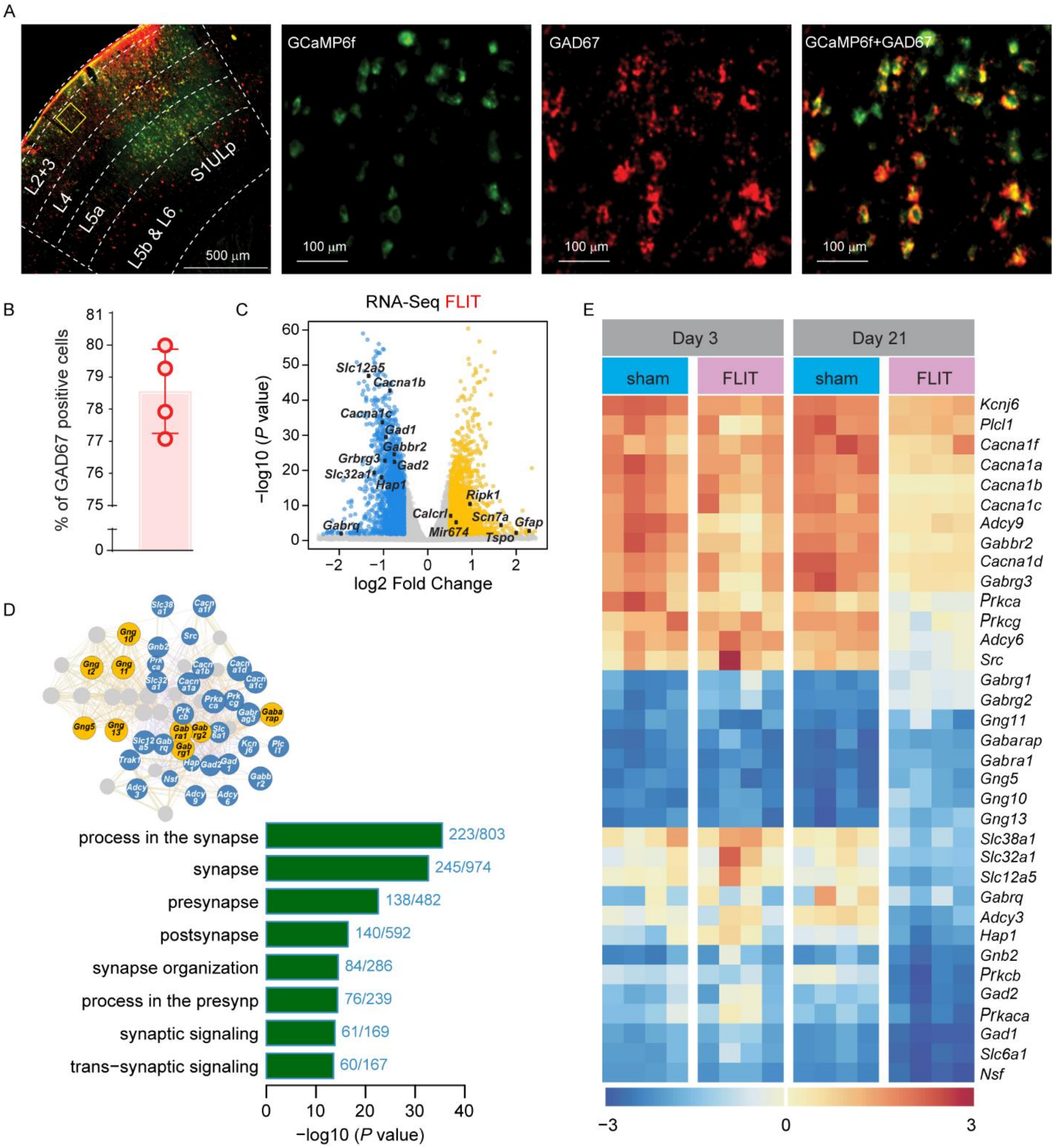

Figure S7. Cortical interneuron targeting and RNA-Seq of FLIT model. (A and B) AAV-Dlx-GCaMP6f expression in cortical interneurons. Mice were sacrificed 28 days after virus injection for brain slices. **(A)** Representative images of GAD67 staining (n = 4). **(B)** percentage of GAD67 positive cells among GCaMP6f expressing cells. **(C)** Interactome of differential regulated genes in FLIT model. Yellow: upregulated FLIT *vs.* sham; blue: downregulated FLIT *vs.* sham; cut-off threshold: 0.5 log2. **(D)** SynGO analysis of synapse-related pathway changes in the FLIT model.

(E) Differentially regulated genes in the FLIT and sham mice ($n = 4$ each group, both normalized to Sham $n = 4$) at days 3 and 21 post surgery. Scale bar represents log2 fold changes.

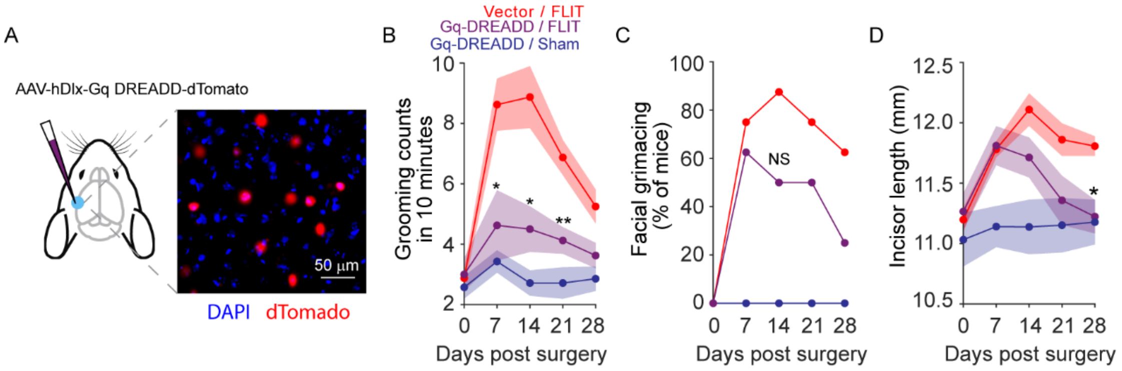

Figure S8. Chemogenetic manipulation of S1 interneurons. (A) Schematic and image of virus injection and expression (scale bar represents 50 μm). **(B)** Facial grooming counts in 10 minutes. Two-way ANOVA test indicates a significant difference among the groups. Post-hoc Bonferroni test was carried out to determine the P value of Vector / FLIT *vs.* Gq-DREADD / FLIT, $*P < 0.05$, $**P < 0.01$. **(C)** Percentage of mice with facial grimacing. Fisher exact test indicates no statistically significant difference among the groups. **(D)** Incisors length at indicated time points. Two-way ANOVA test indicates a significant difference among the groups. Post-hoc Bonferroni test was carried out to determine the P value of Vector / FLIT *vs.* Gq-DREADD / FLIT, $*P < 0.05$.

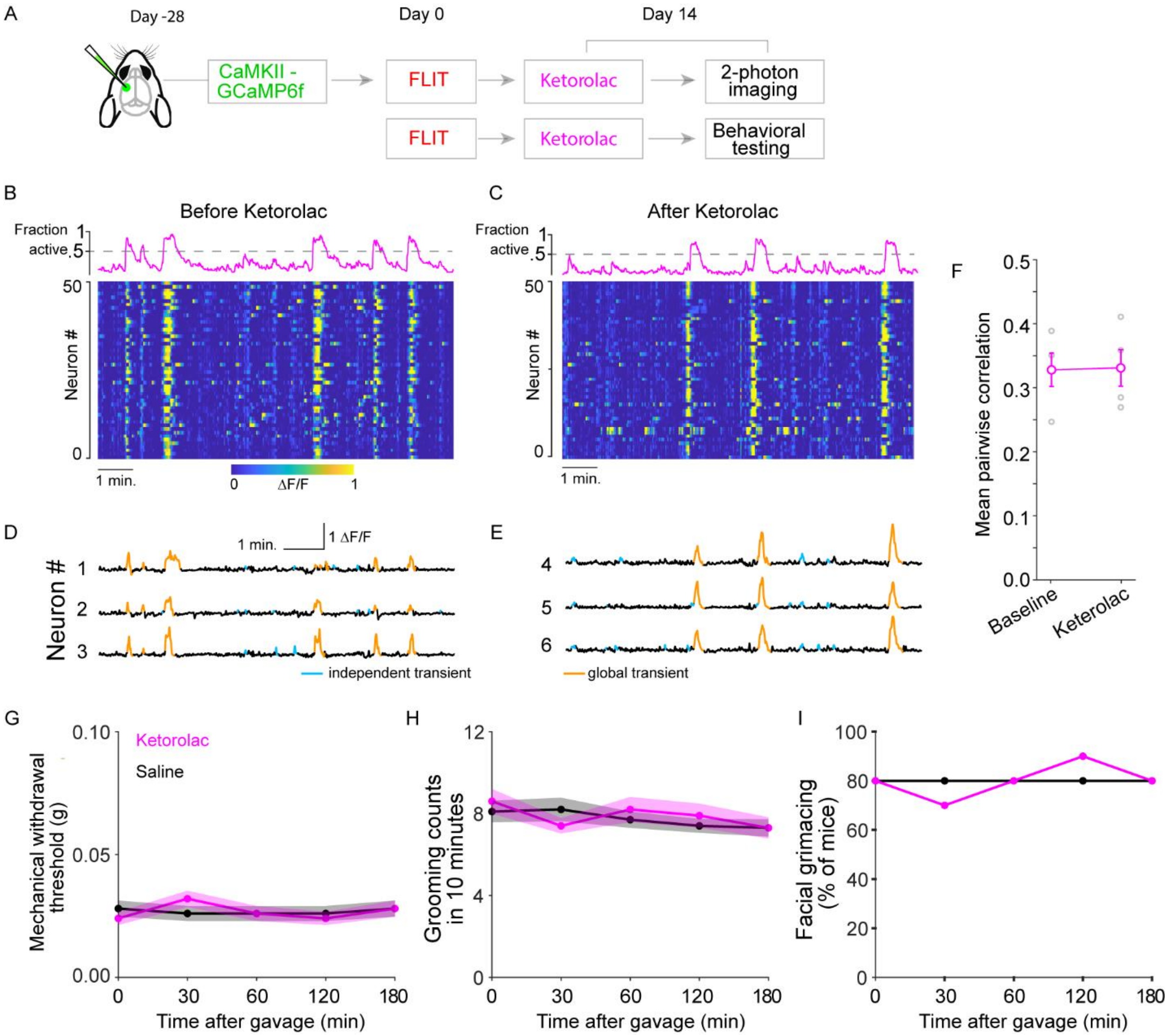

Figure S9. Ketorolac does not alleviate synchronized S1 activity or TN-like behaviors. Fourteen days post FLIT surgery, mice received ketorolac (10 mg/kg, oral gavage) treatment before excitatory neuron calcium imaging (n = 4) or behavioral testing (n = 10). **(A)** Flowchart of ketorolac experiment. **(B and C)** Representative heatmaps; fraction of active neuron plots; and **(D and E)** representative neuronal activity calcium transient traces in the same field of view before (panels B and D) and 60 minutes after (panels C and E) ketorolac administration. **(F)** Mean pairwise correlation before and after ketorolac treatment (Mean ± SEM: before: 0.33 ± 0.03, after: 0.33 ± 0.03, P = 0.93). **(G to I)** Ketorolac treatment does not alleviate TN-like behaviors in FLIT mice. **(G)** Mechanical withdrawal thresholds to von Frey filaments (mean ± SEM). **(H)** Facial grooming counts in 10 minutes at indicated time points. **(I)** Percentage of mice with facial grimaces.

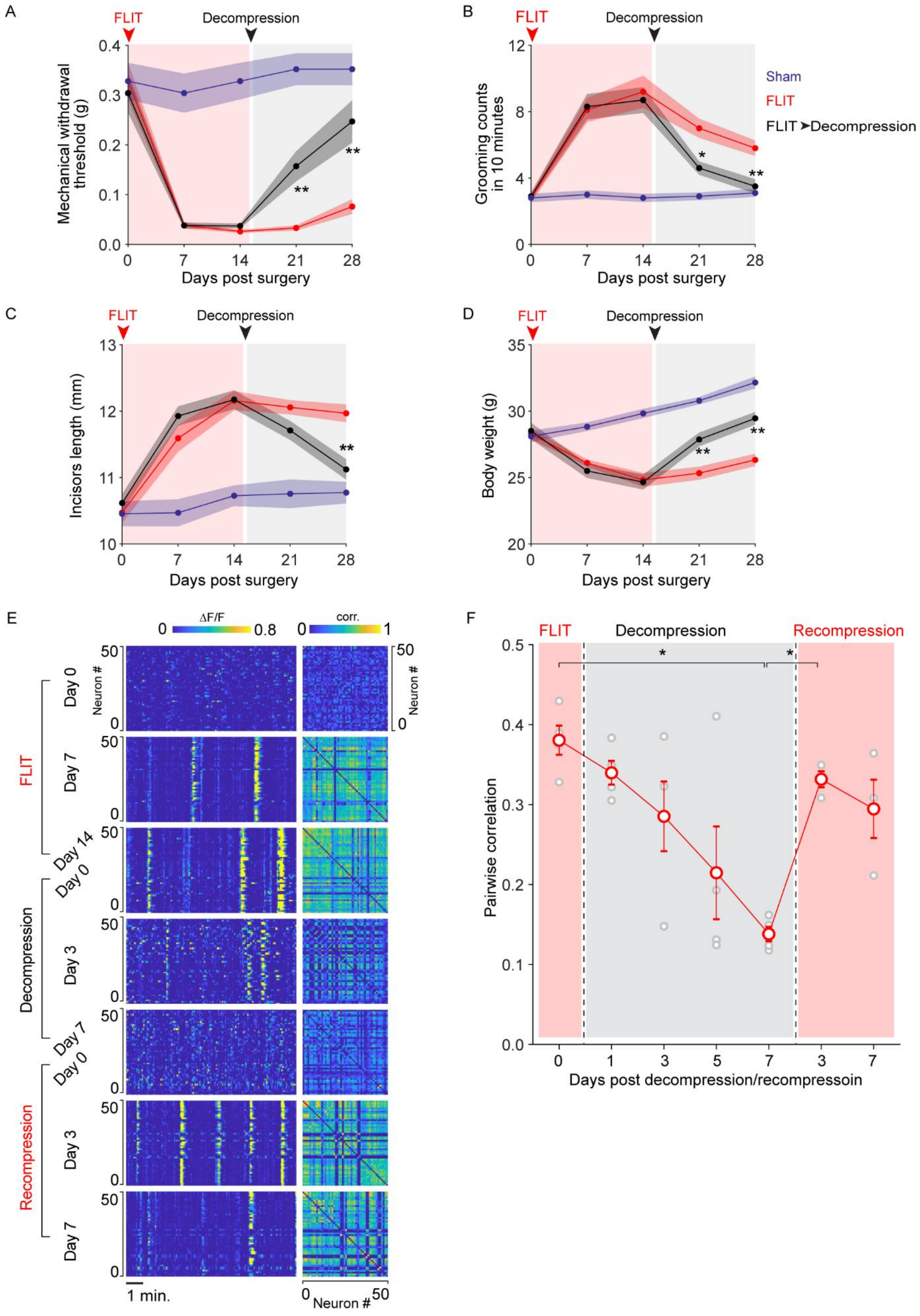
A
FLIT
Decompression
Mechanical withdrawal threshold (g)
Days post surgery
**
**
B
FLIT
Decompression
Grooming counts in 10 minutes
Days post surgery
Sham
FLIT
FLIT ➤Decompression
*
**
C
FLIT
Decompression
Incisors length (mm)
Days post surgery
**
D
FLIT
Decompression
Body weight (g)
Days post surgery
**
**
E
ΔF/F
0
0.8
corr.
0
1
Neuron #
Day 0
Day 7
Day 14
FLIT
Day 0
Day 3
Day 7
Decompression
Day 0
Day 3
Day 7
Recompression
1 min.
Neuron #
F
FLIT
Decompression
Recompression
Pairwise correlation
Days post decompression/recompressoin
*
*

Figure S10. Nerve root decompression and recompression change cortical dynamics and pain-like behavior in FLIT mice. Fourteen days after FLIT surgery, mice (n = 10) received trigeminal nerve decompression. **(A)** Mechanical withdrawal; **(B)** Facial grooming counts in 10 minutes; **(C)** Incisors length; and **(D)** Body weight were compared among the groups. Two-way ANOVA test indicates significant difference was present among the groups, post-hoc Bonferroni test was carried out to determine the P value of FLIT *vs.* FLIT + decompression *$P < 0.05$; **$P < 0.01$. **(E and F)** Reoccurrence of S1 synchronization is induced by recompression of trigeminal nerve root in decompressed FLIT mice (n = 3). Fourteen days post FLIT surgery, mice received decompression by removing Surgifoam through foramen lacerum. Seven days after decompression, mice underwent recompression of trigeminal nerve root. **(E)** Representative S1 excitatory neuronal activity heatmaps and correlation matrices at indicated time points. **(F)** Pairwise correlation of neuronal activity over the time course of the decompression and recompression experiment (One-way ANOVA: $P < 0.05$, Turkey-Kramer post hoc comparison significant for between day 7 decompression and day 3 recompression), *$P < 0.05$. Disclosure: 1) Figure S10E, Day 0 Decompression is the same as Figure 6H pre decomp row, 2) Figure S10E, Decompression Day 7 is the same as Figure 6H post decomp day 7, 3) quantification of data in Figure S10F and Figure 6I share a subset of data points.

Movie S1: Excessive facial grooming in FLIT model.
Movie S2: Paroxysmal asymmetrical facial grimacing in FLIT model.
Movie S3: Synchronized S1 neuronal activity in the same field of view of a FLIT mouse.
